# YY1 downregulation underlies therapeutic response to molecular targeted agents

**DOI:** 10.1038/s41419-024-07239-8

**Published:** 2024-11-27

**Authors:** Shichao Zhou, Jingyu Zang, Mei-Chun Cai, Kaiyan Ye, Jin Liu, Pengfei Ma, Jie Wu, Chenyang Dai, Haijiao Lu, Qing Zhang, Junhong Jiang, Tianqing Chu, Ying Shen, Li Tan, Guanglei Zhuang, Xiaojing Zhao, Lan Wang, Yu Zhuang, Yujie Fu

**Affiliations:** 1 State Key Laboratory of Systems Medicine for Cancer, Department of Thoracic Surgery, Shanghai Cancer Institute, Ren Ji Hospital, Shanghai Jiao Tong University School of Medicine, Shanghai, China; 2 Department of Thoracic Medical Oncology, Zhejiang Cancer Hospital, Hangzhou Institute of Medicine (HIM), Chinese Academy of Sciences, Hangzhou, China; 3 Department of Radiation Oncology, Ren Ji Hospital, Shanghai Jiao Tong University School of Medicine, Shanghai, China; 4 Department of Pathology, The Affiliated Hospital of Qingdao University, Qingdao, China; 5 Department of Thoracic Surgery, Shanghai Pulmonary Hospital, Tongji University School of Medicine, Shanghai, China; 6 Department of Respiratory and Critical Care Medicine, Shanghai Chest Hospital, Shanghai Jiao Tong University School of Medicine, Shanghai, China; 7 Department of Respiratory and Critical Care Medicine, The Fourth Affiliated Hospital of Soochow University, Suzhou, China; 8 Department of Pharmacology and Chemical Biology, Key Laboratory of Cell Differentiation and Apoptosis of Chinese Ministry of Education, Shanghai Jiao Tong University School of Medicine, Shanghai, China; 9 Interdisciplinary Research Center on Biology and Chemistry, Shanghai Institute of Organic Chemistry, Chinese Academy of Sciences, Shanghai, China; 10 Shanghai Key Laboratory of Gynecologic Oncology, Ren Ji Hospital, Shanghai Jiao Tong University School of Medicine, Shanghai, China; 11 Department of Respiratory and Critical Care Medicine, The Affiliated Jiangyin Hospital of Nantong University, Jiangyin, China; 12 Department of Oncology, The Affiliated Hospital of Qingdao University, Qingdao, China

**Keywords:** Targeted therapies, Transcriptional regulatory elements

## Abstract

During targeted treatment, oncogene-addicted tumor cells often evolve from an initial drug-sensitive state through a drug-tolerant persister bottleneck toward the ultimate emergence of drug-resistant clones. The molecular basis underlying this therapy-induced evolutionary trajectory has not yet been completely elucidated. Here, we employed a multifaceted approach and implicated the convergent role of transcription factor Yin Yang 1 (YY1) in the course of diverse targeted kinase inhibitors. Specifically, pharmacological perturbation of the receptor tyrosine kinase (RTK)/mitogen-activated protein kinase (MAPK) pathway resulted in the downregulation of YY1 transcription, which subsequently resumed upon therapeutic escape. Failure to decrease YY1 subverted cytotoxic effects, whereas elimination of residual YY1 maximized anticancer efficacy and forestalled the emergence of drug resistance. Mechanistically, YY1 was uncovered to dictate cell cycle and autophagic programs. Immunohistochemical analysis on a wide spectrum of clinical specimens revealed that YY1 was ubiquitously expressed across lung adenocarcinomas and exhibited anticipated fluctuation in response to corresponding RTK/MAPK inhibition. These findings advance our understanding of targeted cancer management by highlighting YY1 as a determinant node in the context of genotype-directed agents.

## Background

Stepwise progression to drug resistance represents a major challenge in anticancer treatment targeting driver oncoproteins such as EGFR, ALK, KRAS, BRAF, and many others [1]. Initially, presumed lethal therapeutic insults often yield dramatic clinical responses. Nevertheless, a reservoir of drug-tolerant persister cells, commonly termed minimal residual disease, invariably manage to survive and eventually result in tumor relapse [2, 3]. Throughout this evolutionary process, an increasing variety of genetic [4, 5], epigenomic [6, 7], transcriptional [8, 9], translational [10, 11], post-translational [12, 13], and metabolic [14, 15] mechanisms operate to underpin the transition and maintenance of discrete stages, depending on specific cancers and therapies. Such a multifactorial and heterogeneous regulatory landscape poses significant obstacles to effective management and durable control of recalcitrant malignancies [16]. Further in-depth understanding of molecular profiles at different layers and phases may provide new insights into convergent events underlying how neoplastic cells respond and evade targeted agents.

Chromatin accessibility dynamics perhaps constitute one of the most relevant but overlooked genomic characteristics corresponding to the meticulous changes following treatment with targeted therapies [17, 18]. The phenotypic alterations associated with the sequential drug-sensitive, drug-tolerant, and drug-resistant switch probably require widespread remodeling of open chromatin architecture and consequent rewiring of gene expression programs. Indeed, mounting evidence implicates unique chromatin states in the response, survival, and resurgence of cancer cells that are determined by varied epigenetic modifiers [6, 19–23]. However, additional key players involved in this process remain to be elucidated for developing rational therapeutic strategies.

Here, we integrated the assay for transposase-accessible chromatin using sequencing (ATAC-seq) and transcriptome-wide RNA-sequencing (RNA-seq) to identify Yin Yang 1 (YY1) as a pivotal transcription factor in the context of diverse molecular therapeutics targeting receptor tyrosine kinase (RTK)/mitogen-activated protein kinase (MAPK). YY1 was transcriptionally downstream of RTK/MAPK signaling and governed the anticancer effects of pathway blockade by regulating cell cycle and autophagic flux. These mechanistic findings illuminate a general hub underlying drug-induced evolutionary trajectories in oncogene-addicted tumor cells, which holds immense promise for novel intervention opportunities.

## Results

### Characterization of chromatin accessibility dynamics implicates YY1 in EGFR inhibitor response

**Fig. 1:**
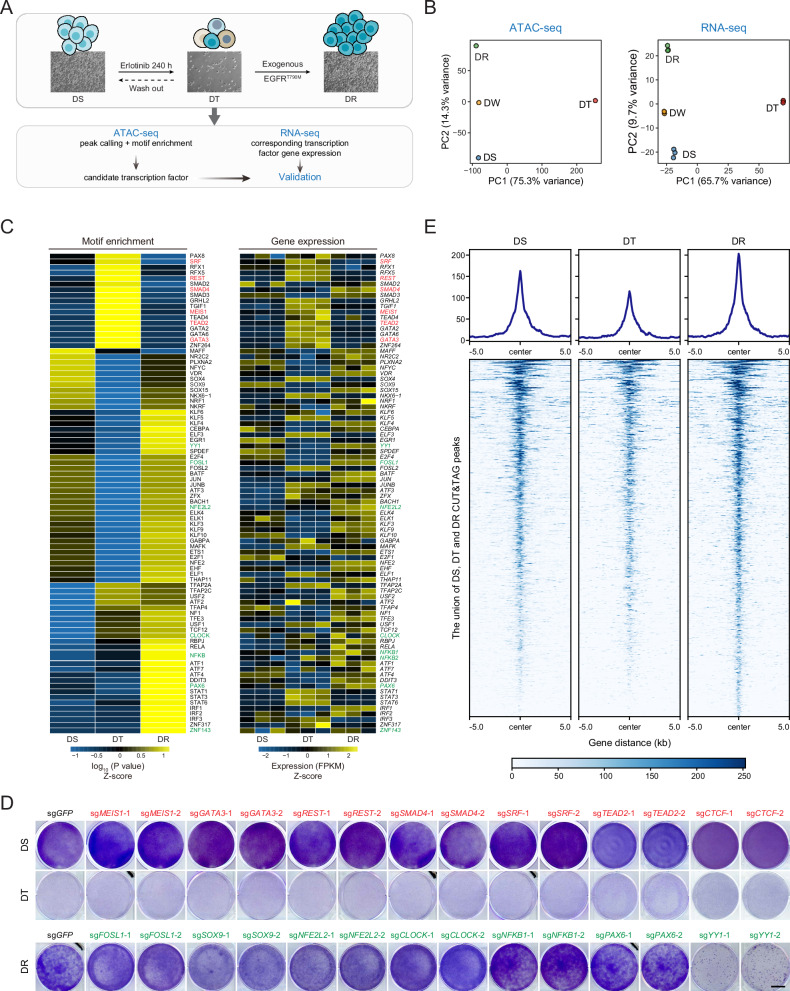
Characterization of chromatin accessibility dynamics implicates YY1 in EGFR inhibitor response. **A** A schematic diagram illustrates the modeling of therapy-induced evolutionary trajectory in PC9 cells and integrated sequencing analyses to identify candidate transcription factors involved in EGFR inhibitor response. **B** Principal component analysis of ATAC-seq and RNA-seq data derived from PC9 cells at the initial drug-sensitive state (DS), drug-tolerant persister state (DT), deliberate drug-withdrawn state (DW), and acquired drug-resistant state (DR). **C** Heatmaps showing dynamic changes of binding motif enrichment (left) and gene expression (right) for candidate transcription factors involved in EGFR inhibitor response. Genes labeled in red displaying upregulation from DS to DT and genes labeled in green displaying upregulation from DT to DR were selected for subsequent experiments. **D** Candidate transcription factors involved in EGFR inhibitor response were each knocked out in parental PC9 cells or PC9 cells with EGFR^T790M^ mutant, and cell viability was assayed by crystal violet staining. Scale bar represents 10 mm. **E** CUT&Tag profiles showing dynamic changes of YY1 occupancy in PC9 cells at the initial drug-sensitive state (DS), drug-tolerant persister state (DT), and acquired drug-resistant state (DR).

We modeled the drug-sensitive (DS), drug-tolerant (DT), and drug-resistant (DR) continuum by using the clinically approved EGFR inhibitor erlotinib to treat a well-studied *EGFR*-mutant lung cancer cell line PC9, which was subjected to ATAC-seq and RNA-seq in parallel (Fig. 1A). Principal component analysis unveiled a comparable pattern of these different states in ATAC-seq and RNA-seq results, with DT cells being clearly separated from DS and DR cells (Fig. 1B). The above observation was confirmed by volcano plots of differential chromatin accessibility (Supplementary Fig. 1A) and gene expression (Supplementary Fig. 1B). ATAC-seq profiling identified seven distinctive clusters of accessible DNA loci (Supplementary Fig. 2A), which were presumably occupied by diverse transcription factors (Supplementary Fig. 2B). In addition, we performed motif enrichment analysis on the entire set of ATAC-seq peaks to infer candidate functional transcription factors and filtered them by querying the corresponding RNA-seq data (Fig. 1C). Based on this integrated approach, a group of transcription factors displaying dynamic changes throughout targeted treatment were selected for a focused CRISPR-Cas9 screen. YY1, an evolutionarily conserved GLI-Krüppel zinc finger transcription factor [24], conferred cell suppression upon genetic depletion (Fig. 1D). We leveraged the CUT&Tag technology (Supplementary Fig. 3A) to verify that genome-wide YY1 binding underwent a marked decrease in DT cells compared to DS cells and was completely restored in DR cells (Fig. 1E). Of note, the gene expression of various putative cancer stem cell markers exhibited inconsistent changes (Supplementary Fig. 3B), suggesting a limited role for cancer stemness. Therefore, YY1 might partially account for kinetic alterations in chromatin openness and serve as a potential regulator of the EGFR inhibitor response.

### EGFR regulates YY1 expression through the MAPK signaling pathway

**Fig. 2:**
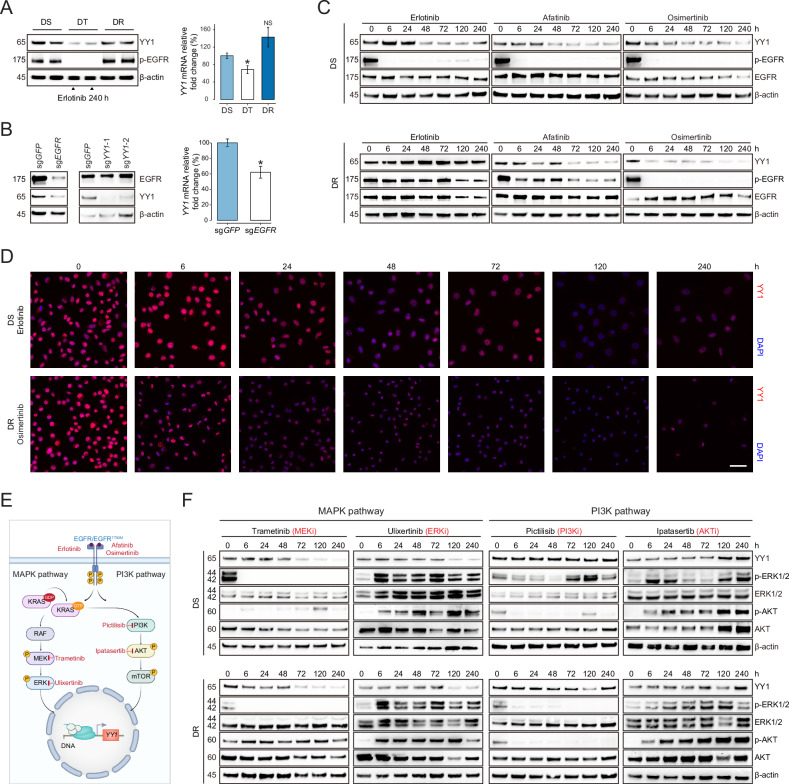
EGFR regulates YY1 expression through the MAPK signaling pathway. **A** Relative YY1 protein and *YY1* mRNA levels in PC9 cells at the initial drug-sensitive state (DS), drug-tolerant persister state (DT), and acquired drug-resistant state (DR). **B** *EGFR* or *YY1* gene was knocked out in PC9 cells using the CRISPR-Cas9 system. The indicated proteins were analyzed by immunoblotting, and the relative expression of *YY1* mRNA was measured by quantitative PCR analysis. **C** Parental PC9 cells or PC9 cells with EGFR^T790M^ mutant were treated with erlotinib, afatinib or osimertinib in a time course manner, and the indicated proteins were analyzed by immunoblotting. **D** Immunofluorescence staining of YY1 (red) in parental PC9 cells or PC9 cells with EGFR^T790M^ mutant in the presence of erlotinib or osimertinib treatment, respectively. Cell nuclei were counterstained with DAPI (blue). Scale bar represents 50 μm. **E** A schematic diagram illustrating the EGFR signaling pathway and corresponding kinase inhibitors that may modulate YY1 expression. **F** Parental PC9 cells (DS) or PC9 cells with EGFRT790M mutant (DR) were treated with MAPK pathway inhibitors (trametinib and ulixertinib) or PI3K pathway inhibitors (pictilisib and ipatasertib) in a time course manner, and the indicated proteins were analyzed by immunoblotting.

Correlated with fluctuating YY1 occupancy following EGFR inhibitor treatment, YY1 protein abundance notably declined in DT cells relative to DS or DR cells, which was ascribable to altered *YY1* gene transcription (Fig. 2A). Corroborating the pharmacological evidence, genetic *EGFR* depletion likewise resulted in YY1 downregulation at both mRNA and protein levels, whereas *YY1* ablation did not affect EGFR expression (Fig. 2B). Moreover, immunoblotting assays revealed that YY1 intensity fully emulated EGFR phosphorylation when PC9 cells were exposed to the first-, second-, or third-generation EGFR inhibitors in a time course fashion (Fig. 2C). Similar associations were recapitulated in various *EGFR*-mutant lung cancer cell lines including HCC827, NCI-H1975, and NCI-H820 (Supplementary Fig. 4A). Immunofluorescence staining indicated predominant nuclear localization of YY1, which showed diminished signals consequent to EGFR blockade (Fig. 2D). MAPK and PI3K signaling cascades represented two major pathways downstream of EGFR kinase to possibly regulate YY1 expression. We sought to dissect their specific contributions by administering trametinib (an MEK inhibitor), ulixertinib (an ERK inhibitor), pictilisib (a PI3K inhibitor), or ipatasertib (an AKT inhibitor) to block either axis (Fig. 2E). In PC9 cells, applying trametinib or ulixertinib was sufficient to induce YY1 downregulation while pictilisib or ipatasertib had no equivalent effects (Fig. 2F), supporting an exclusive role of MAPK but not PI3K pathway. Such a notion was substantiated by validation experiments in HCC827, NCI-H1975, and NCI-H820 cells (Supplementary Fig. 4B). Analogous to erlotinib, trametinib also attenuated YY1 mRNA expression (Supplementary Fig. 4C). Thus, our data unambiguously proved that EGFR regulated YY1 expression through the MAPK signaling pathway. Within the MAPK pathway, we further tested a number of candidate transcription factors that were predicted to bind to the YY1 promoter, including ETS1, CREB1, SRF, ELK1, ETV1, SP1, EGR1, RREB1, MAZ, and CEBPA. However, none of them showed notable regulatory effects on YY1 protein levels based on siRNA knockdown experiments (Supplementary Fig. 4D). Given the multitude and complexity of transcription factors downstream of the MAPK pathway, larger-scale screening might be needed, or alternatively, multiple transcription factors could compensate for each other to control YY1 expression.

### RTK/MAPK inhibition downregulates YY1 in diverse oncogene-addicted cancer models

**Fig. 3:**
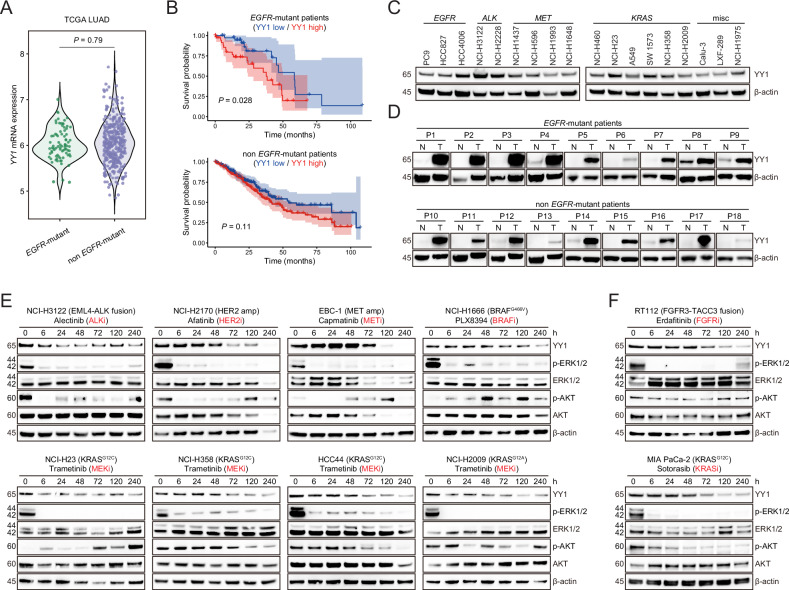
RTK/MAPK inhibition downregulates YY1 in diverse oncogene-addicted cancer models. **A** Violin plots indicating the relative expression of *YY1* mRNA in TCGA lung adenocarcinomas (LUAD) with or without *EGFR* mutations. **B** Kaplan–Meier plots illustrating associations between *YY1* expression and overall survival in 80 TCGA LUAD patients with *EGFR* mutations (top) and 427 TCGA LUAD patients without *EGFR* mutations (bottom). *P*-values are based on log-rank tests. The light red or blue shading indicates 95% confidence interval. **C** YY1 protein was analyzed by immunoblotting in a panel of lung cancer cell lines with diverse oncogenic alterations. **D** YY1 protein was analyzed by immunoblotting in normal lung tissues and paired tumor lesions from human lung adenocarcinomas with or without *EGFR* mutations. **E** Lung cancer cell lines with diverse oncogenic alterations were treated with genotype-matched kinase inhibitors in a time course manner, and the indicated proteins were analyzed by immunoblotting. **F** A bladder cancer cell line with *FGFR3* rearrangement and a pancreatic cancer cell line with *KRAS* mutation were treated with genotype-matched kinase inhibitors in a time course manner, and the indicated proteins were analyzed by immunoblotting.

We reasoned that YY1 function might not be restricted to *EGFR*-mutant lung cancer. Indeed, *YY1* gene expression was unrelated to EGFR status in lung adenocarcinoma from the Cancer Genome Atlas (Fig. 3A). In addition, higher levels of *YY1* mRNA tended to predict inferior patient survival irrespective of *EGFR* alterations (Fig. 3B). At the protein level, immunoblotting assays illustrated that YY1 was readily detectable in a wide spectrum of oncogene-driven lung cancer cell lines (Fig. 3C), as well as in most primary tumor samples regardless of *EGFR* mutation (Fig. 3D). Based on these observations, a panel of lung cancer cell lines bearing heterogeneous RTK/MAPK aberrations was assembled and treated with corresponding kinase inhibitors. YY1 abundance was appreciably decreased along with ERK repression in a time-dependent manner (Fig. 3E). We then extended our investigation to other cancer types with RTK/MAPK pathway dysregulation. Two examples, erdafitinib (FGFR inhibitor) and sotorasib (KRAS G12C inhibitor) impaired YY1 expression in genotype-matched bladder cancer cell line RT112 and pancreatic cancer cell line MIA PaCa-2, respectively (Fig. 3F). We concluded that RTK/MAPK inhibition downregulated YY1 in diverse oncogene-addicted cancer models.

### YY1 depletion underlies therapeutic effects of molecular targeted agents

**Fig. 4:**
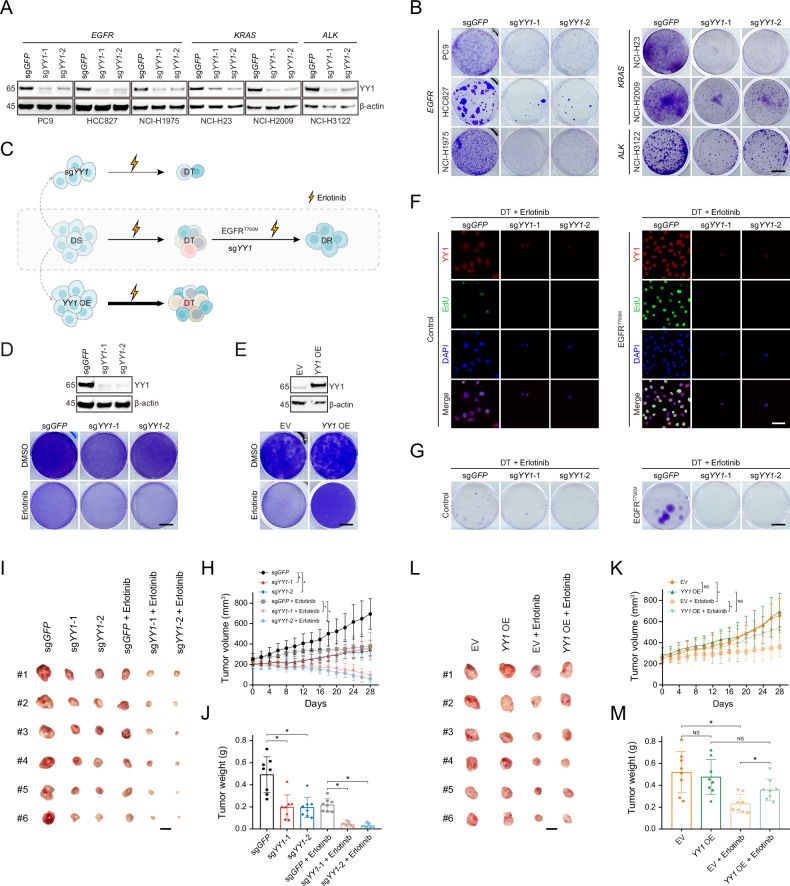
YY1 depletion underlies the therapeutic effects of molecular targeted agents. **A** *YY1* was knocked out in *EGFR*-mutant, *KRAS*-altered, or *ALK*-rearranged lung cancer cell lines with two independent sgRNAs using the CRISPR-Cas9 system, and YY1 protein was analyzed by immunoblotting. **B** *YY1* was knocked out in *EGFR*-mutant, *KRAS*-altered, or *ALK*-rearranged lung cancer cell lines with two independent sgRNAs using the CRISPR-Cas9 system, and cell viability was assessed by crystal violet staining. Scale bar represents 10 mm. **C** A schematic diagram illustrates the genetic perturbation of *YY1* or *EGFR* in PC9 cells at different states. Top and bottom: *YY1* was knocked out or overexpressed in PC9 cells at the initial drug-sensitive state (DS) and then cells were treated with erlotinib to induce drug-tolerant persister state (DT); middle, PC9 cells were treated with erlotinib to form drug-tolerant persister state (DT), and *YY1* or *EGFR* was manipulated as indicated to track acquired drug-resistant state (DR). **D** *YY1* was knocked out in PC9 cells, and YY1 protein was analyzed by immunoblotting. EGFR inhibitor response upon *YY1* depletion was assessed by crystal violet staining. Scale bar represents 10 mm. **E** *YY1* was overexpressed in PC9 cells, and YY1 protein was analyzed by immunoblotting. EGFR inhibitor response upon *YY1* overexpression was assessed by crystal violet staining. Scale bar represents 10 mm. **F** *YY1* was knocked out in PC9-derived drug-tolerant (DT) cells with (left) or without (right) EGFR^T790M^ mutant, followed by immunofluorescence staining of YY1 (red) and EdU (green). Cell nuclei were counterstained with DAPI (blue). Scale bar represents 50 µm. **G** *YY1* was knocked out in PC9-derived drug-tolerant (DT) cells with (left) or without (right) EGFR^T790M^ mutant and cell viability was assessed by crystal violet staining. Scale bar represents 10 mm. **H** Tumor growth curves of *YY1*-depleted PC9 xenografts that were treated with vehicle or erlotinib. Each line represents the mean tumor volume of the respective group. Error bars indicate standard deviation (8 mice/group). *P < 0.05, unpaired Student’s *t*-test. **I** Representative images of PC9 xenografts with or without *YY1* knockout. The xenografts were treated with vehicle (30% PEG 300, 5% Tween 80) or erlotinib (5 mg/kg/day). Scale bar represents 10 mm. **J** Tumor weights of *YY1*-depleted PC9 xenografts that were treated with vehicle or erlotinib. Error bars indicate standard deviation (8 mice/group). *P < 0.05, unpaired Student’s *t*-test. **K** Tumor growth curves of *YY1*-overexpressed PC9 xenografts that were treated with vehicle or erlotinib. Each line represents the mean tumor volume of the respective group. Error bars indicate standard deviation (8 mice/group). *P < 0.05, unpaired Student’s *t*-test. NS denotes not significant. **L** Representative images of PC9 xenografts with or without *YY1* overexpression. The xenografts were treated with vehicle (30% PEG 300, 5% Tween 80) or erlotinib (5 mg/kg/day). Scale bar represents 10 mm. **M** Tumor weights of *YY1*- overexpressed PC9 xenografts that were treated with vehicle or erlotinib. Error bars indicate standard deviation (8 mice/group). *P < 0.05, unpaired Student’s *t*-test. NS denotes not significant.

We wondered whether YY1 exerted functional activities, especially in the context of molecular targeted regimens. Initially, lentiviral CRISPR-Cas9 technology was employed to knockout *YY1* in multiple *EGFR*-mutant, *KRAS*-altered, or *ALK*-rearranged lung cancer cell lines using two independent sgRNAs (Fig. 4A), and cell viability was significantly inhibited without exception (Fig. 4B). Subsequently, we genetically manipulated *YY1* expression at discrete phases of EGFR inhibitor treatment and assessed its impact on therapeutic outcomes (Fig. 4C). First, *YY1* ablation in DS cells improved drug sensitivity by preventing DT emergence in PC9 (Fig. 4D), as well as HCC827, NCI-H1975, and NCI-H820 (Supplementary Fig. 5A). Second, *YY1* overexpression in DS cells antagonized the drug response by accelerating DT formation in PC9 (Fig. 4E), as well as HCC827, NCI-H1975, and NCI-H820 (Supplementary Fig. 5B). Third, *YY1* knockout in DT cells, leading to drastically compromised proliferation estimated by EdU incorporation assays (Fig. 4F), eliminated residual colonies and overcame DR expansion (Fig. 4G). Finally, we conducted in vivo experiments to confirm the in vitro findings. Upon *YY1* loss, PC9 xenograft growth was slowed and more susceptible to erlotinib inhibition (Fig. 4H), yielding reduced tumor masses (Fig. 4I) with less weight (Fig. 4J). In contrast, exogenous *YY1* expression attenuated erlotinib efficacy (Fig. 4K), as exemplified by larger tumor masses (Fig. 4L) with gained weight (Fig. 4M). Collectively, these studies suggested that YY1 depletion underlay the therapeutic effects of molecular targeted agents.

### YY1 dictates cell cycle and autophagic programs

**Fig. 5:**
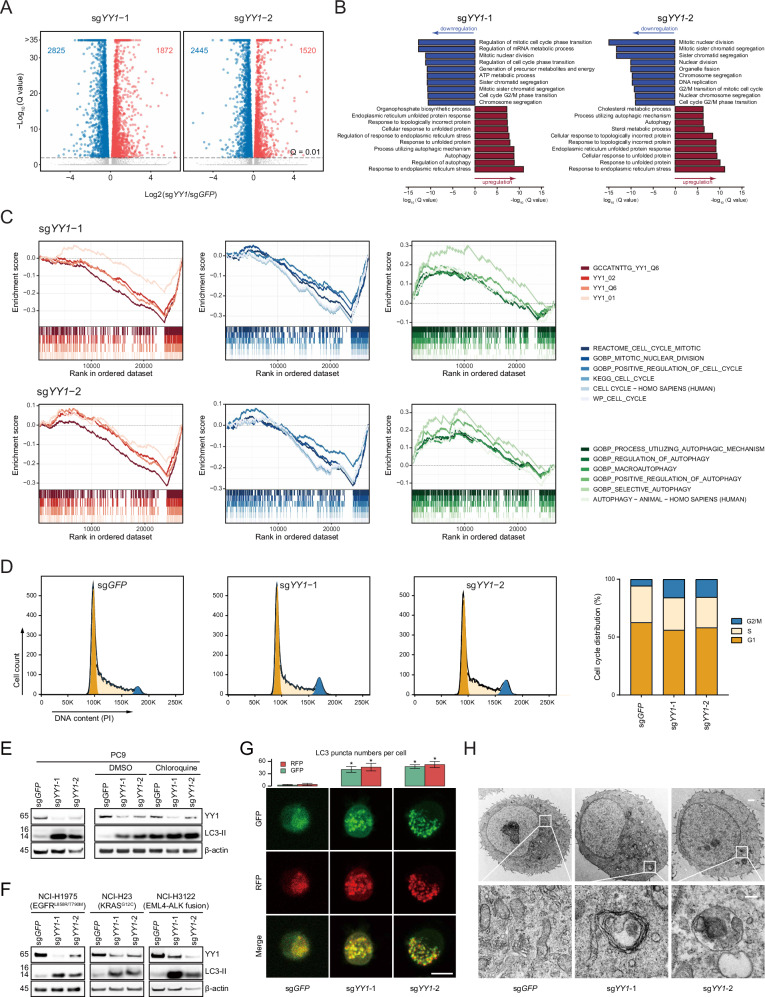
YY1 dictates cell cycle and autophagic programs. **A** Volcano plot showing differentially expressed genes after *YY1* knockout by two independent sgRNAs in PC9 cells. **B** Gene ontology categories overrepresented in differentially expressed genes upon *YY1* knockout. Red arrows indicate upregulated pathways and blue arrows indicate downregulated pathways. Q value represents the adjusted *P* value. **C** Enrichment plots from gene set enrichment analysis (GSEA) of YY1-related (red), cell cycle-related (blue), and autophagy-related (green) gene signatures. All GSEA results were statistically significant (FDR < 0.05). **D** Cell cycle analysis of YY1-depleted PC9 cells by flow cytometry and bar graphs showing the distribution of the cell cycle phases. **E** *YY1* gene was knocked out in PC9 cells, and the LC3 protein was analyzed by immunoblotting in the presence or absence of chloroquine, a late-stage autophagy inhibitor. **F** *YY1* gene was knocked out in NCI-H1975, NCI-H23, or NCI-H3122 cells, and the LC3 protein was analyzed by immunoblotting. **G** *YY1*-depleted PC9 cells were transfected with the RFP-GFP-LC3 plasmid and analyzed by confocal microscopy. Yellow puncta indicate autophagosomes while red puncta indicate autolysosomes. The top panel shows the quantification of LC3 puncta per cell. *P < 0.05, unpaired Student’s *t*-test. Scale bar represents 10 µm. **H** Representative images of transmission electron microscopy demonstrating abnormal accumulation of autophagosomes and autolysosomes in *YY1*-depleted PC9 cells. Scale bar of the upper images represents 1 µm and the scale bar of the lower images represents 0.2 µm.

To determine the mechanistic underpinnings of YY1 function, RNA-seq was performed to compare *YY1*-null and parental PC9 cells (Supplementary Fig. 6A). As a transcription factor, *YY1* knockout resulted in a considerable number of differentially expressed genes (DEGs) (Fig. 5A; Supplementary Fig. 6B). There was a significant positive correlation between fold changes induced by two sgRNAs at the transcriptome-wide scale (Supplementary Fig. 6C), and Venn diagram illustrated that 2835 out of 5811 DEGs (48.8%) were shared (Supplementary Fig. 6D). Gene ontology annotation pinpointed that cell cycle-related terms were downregulated while stress- and autophagy-related pathways were upregulated (Fig. 5B). Gene set enrichment analysis (Fig. 5C) and Metascape algorithm (Supplementary Fig. 7A) validated the enriched biological processes associated with *YY1* ablation. As the experimental proof, cell cycle examination by flow cytometry uncovered that *YY1*-depleted PC9 cells were arrested at the G2/M phase (Fig. 5D). A closer inspection on the autophagic machinery found that in the absence of *YY1* [25, 26], many regulators were widely altered (Supplementary Fig. 7B), among which early autophagy-related genes were generally upregulated whereas late autophagy-related genes were frequently downregulated (Supplementary Fig. 7C). LC3-II, a commonly used autophagy indicator, was markedly accumulated in PC9 (Fig. 5E), as well as NCI-H1975, NCI-H23, and NCI-H3122 (Fig. 5F). Of note, similar phenomenon was observed in chloroquine-treated PC9 cells, in line with autophagic flux suppression. Consistently, the tandem RFP-GFP-LC3 probe revealed a rise in autophagosomes (RFP^+^GFP^+^ puncta) rather than acidified autolysosomes (RFP^+^GFP^-^ puncta) (Fig. 5G). Morphometric transmission electron microscopy identified abundant autophagosomes but few autolysosomes upon *YY1* depletion, suggestive of deficient autophagosome-lysosome fusion (Fig. 5H). These data supported that YY1 might affect therapeutic response to molecular targeted agents by dictating cell cycle and autophagic programs [27–31].

### YY1 exhibits anticipated fluctuation following targeted treatment in lung cancer patients

**Fig. 6:**
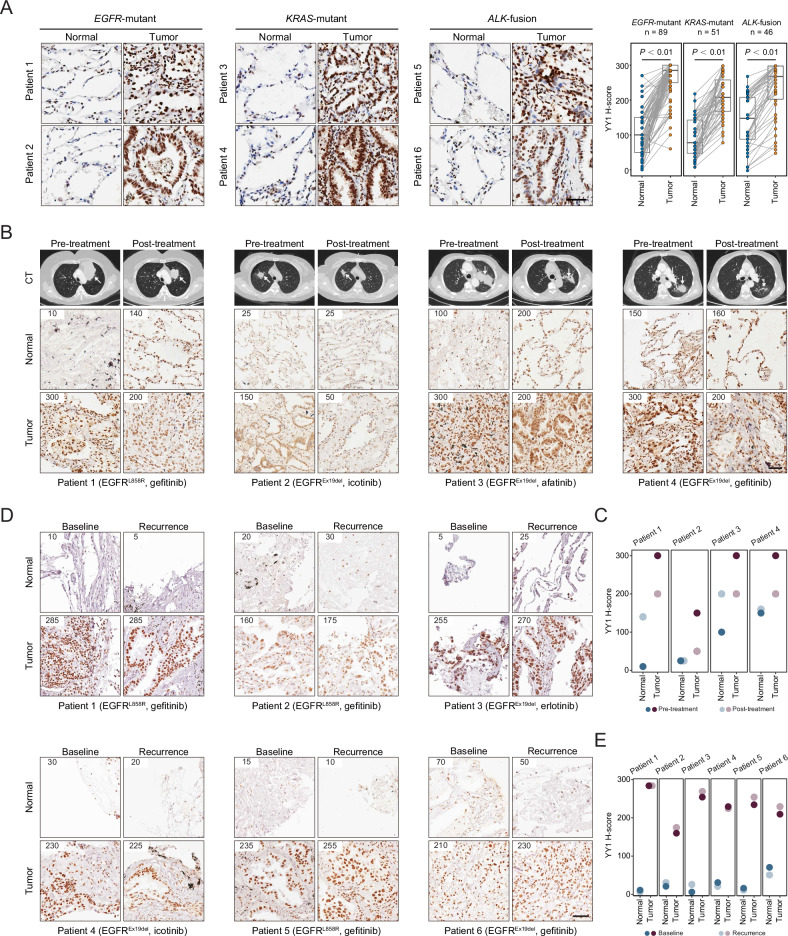
YY1 exhibits anticipated fluctuation following targeted treatment in lung cancer patients. **A** Immunohistochemistry staining of YY1 in a cohort of 186 patients affected by *EGFR*-mutant, *KRAS*-altered, or *ALK*-rearranged lung cancer. Blue dots indicate normal lung tissues, and orange dots indicate paired tumor lesions. *P*-values are based on paired Student’s *t*-tests. Scale bar represents 50 µm. **B** Computed tomography (CT) images and representative YY1 immunohistochemistry staining in *EGFR*-mutant lung adenocarcinomas before and after neoadjuvant EGFR inhibitors. The H-score system was used for immunohistochemical quantification. Scale bar represents 50 µm. **C** Dot plots indicating intensity changes of YY1 staining in normal lung tissues and paired tumor lesions before and after neoadjuvant targeted therapy. **D** Representative YY1 immunohistochemistry staining in *EGFR*-mutant lung adenocarcinomas at baseline and upon disease recurrence following first-line EGFR inhibitor treatment. The H-score system was used for immunohistochemical quantification. Scale bar represents 50 µm. **E** Dot plots indicating intensity changes of YY1 staining in normal lung tissues and paired tumor lesions at baseline and upon disease recurrence following first-line EGFR inhibitor treatment.

To investigate the clinical relevance of YY1 expression, three cohorts of lung adenocarcinoma were collected. First, immunohistochemical interrogation of 89 *EGFR*-mutant, 51 *KRAS*-altered, and 46 *ALK*-rearranged lung cancer specimens unveiled that YY1 was ubiquitously expressed in malignant cells at an elevated level relative to the normal epithelium (Fig. 6A; Supplementary Table 1). Second, YY1 was evaluated in four lung cancer patients who harbored somatic *EGFR* mutations and received off-label neoadjuvant targeted therapies (Fig. 6B; Supplementary Table 2). Although YY1 was highly expressed in tumor lesions compared to adjacent normal tissues at baseline, a consistent reduction was observed in post-treatment versus pre-treatment samples (Fig. 6C). Third, YY1 was analyzed in six cases of advanced *EGFR*-mutant lung adenocarcinoma with disease relapse following first-line EGFR inhibitor treatment (Fig. 6D; Supplementary Table 3), which showed that YY1 levels rebounded to baseline in recurrent tumors (Fig. 6E). Taken together, YY1 was ubiquitously expressed and exhibited anticipated fluctuation following targeted treatment in lung cancer patients.

## Discussion

This study presented a global map of chromatin accessibility and transcriptomic landscape during EGFR perturbation. Integrated bioinformatics and functional analyses highlighted a model implicating the transcription factor YY1 as a master regulator downstream of RTK/MAPK signaling. We showed how YY1 might govern antineoplastic output by dictating cell cycle and autophagic programs. These discoveries revealed an exciting and previously unappreciated component in charge of therapeutic response to molecular targeted agents and pointed to future avenues for improving the magnitude and duration of clinical benefit.

Despite the proven significance and current limitations of targeted treatment to reduce cancer mortality, our understanding of therapy-induced evolutionary trajectory remains surprisingly incomplete [32]. Recent literature has individually focused on persister or refractory cells, both constituting major causes of disease relapse [2, 3, 16]. Using *EGFR*-mutant lung cancer as a representative system, we extended prior investigations in two dimensions to address this unsolved fundamental challenge. First, the whole progressive course upon drug intervention was modeled across the continuum of drug-sensitive, drug-tolerant, and drug-resistant phases to approximate a typical scenario in clinic. Second, ATAC-seq and RNA-seq were combined to thoroughly profile the molecular dynamics at different layers. Such a comprehensive interrogation allowed to identify that YY1 underwent kinetic alterations of genomic occupancy and gene expression. Of special importance, YY1 was found to be not only modulated by EGFR but also broadly controlled by RTK/MAPK signaling at the transcriptional level, although the downstream transcription factors require continued research. Thus, our research pinpointed a universal program operating in the course of diverse kinase inhibitors targeting the RTK/MAPK cascade to treat oncogene-driven malignancies. It is noteworthy that YY1 could not be fully eliminated even with prolonged drug exposure, implying that additional regulatory modes may exist and warrant further exploration.

YY1 belongs to the GLI-Krüppel family of zinc finger transcription factors [24]. It is highly expressed in many tumor types and regulates numerous genes involved in hallmarks of cancer including uncontrolled cell proliferation, invasive behavior, metabolic reprogramming, and drug resistance [33–39]. Along this line, our data unraveled that cell cycle and autophagic flux, among a plethora of other pathways, were evidently impacted by *YY1* ablation, which offered mechanistic interpretations of YY1 function in anticancer treatment. Moreover, YY1 can act as a transcriptional activator or repressor depending on the context. Indeed, we discovered that YY1 exerted dichotomous effects on early or late autophagy-related genes, aggregately leading to defective autophagic progression. Beyond its role as a traditional transcription factor, YY1 has been reported to promiscuously interact with cofactors and chromatin modifiers [40], mediate enhancer-promoter looping [41], and possibly form phase-separated condensates [42]. Therefore, its exact mechanism of action underlying targeted therapeutics needs future in-depth dissection.

One critical question concerns whether preclinical observations recapitulate the clinical reality in cancer patients. Notably, we collected three cohorts of lung adenocarcinoma to resolve this issue. At baseline, YY1 was ubiquitously detected across a total of 186 primary tumors spanning *EGFR*-mutant, *KRAS*-altered, and *ALK*-rearranged specimens. In four *EGFR*-mutant subjects receiving EGFR inhibitors and longitudinal sampling, there was a consistent decrease of YY1 protein in paired post-treatment versus pre-treatment sections. Finally, YY1 expression was invariably resumed upon disease recurrence following first-line EGFR inhibitor treatment in six *EGFR*-mutant patients. Collectively, we demonstrated that YY1 levels determined the cytotoxic response to molecular targeted agents, providing a promising biomarker for evaluating drug effectiveness. In addition, our work establishes a sound rationale to develop combination regimens that eradicate YY1 remnants at drug-tolerant states or prohibit YY1 recovery during drug-resistant transit. Considering that oncogenic activation of the EGFR pathway results in an immune-inert phenotype and recent literature has implicated a potential role of YY1 in mediating tumor immune escape [36, 43–46], these conclusions may also be tentatively extrapolated to cancer immunotherapy.

## Materials and methods

### Cell culture and reagents

Tumor cell lines and HEK293T cells were originally obtained from the American Type Culture Collection (ATCC) or Japanese Collection of Research Bioresources Cell Bank (JCRB), where mycoplasma contamination was routinely tested and cell identity was monitored by short tandem repeat (STR) profiling. Drug-tolerant persister cells were generated by continuous treatment with 1 μM erlotinib for 10 days, according to protocols described previously [6]. Drug-resistant cells were generated by overexpressing the EGFR^T790M^ mutant in PC9 cells and then maintained in 1 μM erlotinib. Cells were cultured in RPMI1640 (Life Technologies) supplemented with 10% fetal bovine serum (Gibco), l-glutamine (2 mM), penicillin (100 units/mL), and streptomycin (100 μg/mL). Small-molecule inhibitors were purchased from Selleck Chemicals or MedChemExpress and reconstituted in DMSO (Sigma–Aldrich) at a stock concentration of 10 mM. The following inhibitors were used at these final concentrations unless otherwise indicated: erlotinib (1 μM), afatinib (4 μM), osimertinib (4 μM), trametinib (5 μM), ulixertinib (1 μM), pictilisib (1 μM), ipatasertib (1 μM), alectinib (5 μM), capmatinib (4 μM), PLX8349 (4 μM), erdafitinib (2 μM), sotorasib (1 μM), chloroquine (50 μM). For visualization, cells were fixed with formalin and stained with crystal violet.

### Plasmids, siRNA, and sgRNAs

Plasmids for gene overexpression were constructed using the Gibson Assembly Cloning Kit (New England Biolabs) and Gateway Cloning System (Invitrogen). *EGFR* mutations were generated using the Q5 Site-Directed Mutagenesis Kit (New England Biolabs) and verified by Sanger sequencing. All siRNAs were used at a final concentration of 25 nM and transfected into cells with Lipofectamine 2000 reagent according to the manufacturer’s instructions (Thermo Fisher Scientific). The CRISPR-Cas9 system was employed to knockout indicated genes. The primers used for cloning and the sgRNA sequences are provided in Supplementary Table 4.

### Virus production and cell infection

For virus production, HEK293T cells in a 10-cm dish were co-transfected with 5 µg of lentiviral constructs, 5 μg of plasmid Δ8.9, and 3 μg of plasmid VSVG using Lipofectamine 2000. Cells were incubated at 37 °C and the medium was replaced with fresh complete medium after 12 h. Virus-containing medium was collected 48-72 h after transfection and supplemented with 8 μg/mL polybrene (Fluka) to infect target cells in 6-well dishes. Infected cells were selected with 2–5 μg/mL puromycin or blasticidin for one week.

### Western blotting analysis

Cells were lysed in RIPA buffer (50 mM Tris pH 7.4, 150 mM NaCl, 1% NP-40, 0.1% SDS, 2 μM EDTA) containing protease inhibitors (Roche) and phosphatase inhibitors (Roche). Protein concentrations were quantified using Pierce BCA Protein Assay Kit (Thermo Fisher Scientific). Cell lysates (~20 μg protein) were subjected to SDS-PAGE (Invitrogen) and Western blotting. The following primary antibodies were used: YY1 (ab109237, Abcam), phospho-EGFR (Y1068) (#48576, Cell Signaling Technology), EGFR (#4267, Cell Signaling Technology), phospho-ERK (T202/Y204) (#9106, Cell Signaling Technology), ERK (#4695, Cell Signaling Technology), phospho-AKT (T308) (#2965, Cell Signaling Technology), AKT (#2966, Cell Signaling Technology), LC3-II (#12741, Cell Signaling Technology), β-actin (#5125, Cell Signaling Technology).

### Immunofluorescence staining

Cells were fixed with 4% paraformaldehyde for 15 min, and permeabilized with 0.1% Triton X-100 in PBS for 10 min. After three PBS washes, cells were blocked with 2% BSA in PBS for 30 min at room temperature (RT), and incubated with primary antibodies against YY1 (#66281-1-Ig, Proteintech) diluted in 2% BSA at 4 °C overnight. Cells were incubated with Alexa Fluor 488-labeled anti-mouse IgG (A11029, Invitrogen) and Alexa Fluor 594-labeled anti-rabbit IgG (A11037, Invitrogen) for 1 h at RT in the dark, followed by 4′,6-diamidino-2-phenylindole (DAPI) (Invitrogen) counterstaining for 5 min. The staining images were acquired using a confocal laser scanning microscope (Leica). For RFP-GFP-LC3 analysis, PC9 cells were transfected with 2 μg of tandem monomeric RFP-GFP-LC3 plasmid for 48 h and subjected to the indicated treatment. For evaluating tandem fluorescent LC3 puncta, the cells were rinsed once with PBS followed by confocal microscopy (Leica) analysis. The number of LC3 puncta per cell was counted using Image J (v 2.1.0).

### EdU incorporation assay

The EdU (5-ethynyl-2′-deoxyuridine) incorporation assay was performed with the Cell-Light^TM^ EdU DNA Cell Proliferation Kit (RiboBio) according to the manufacturer’s protocol. Briefly, cells were treated as indicated, incubated with EdU (50 μM) for 2 h, and fixed in 4% paraformaldehyde at RT for 30 min. The cells were stained with ApolloGreen fluorescent dye, followed by incubation with Hoechst reaction solution. Stained samples were viewed under a fluorescence microscope (Leica).

### Patient samples

Human samples were obtained in accordance with the ethical guidelines of the U.S. Common Rule, and the study was approved by the Ethics Committee of Ren Ji Hospital. Written informed consent was acquired from all patients. Paired lung adenocarcinoma samples were collected before and after neoadjuvant targeted therapy, or at baseline and upon disease recurrence following first-line EGFR inhibitor treatment. In addition, we assembled two cohorts of treatment-naïve lung adenocarcinomas. One cohort contained 18 patients, whose tissue specimens were subjected to Western blotting analysis. The other cohort contained 186 patients with driver gene mutations for immunohistochemical staining of the formalin-fixed and paraffin-embedded (FFPE) sections.

### Immunohistochemistry staining

The tissue slides were baked, dewaxed with xylene, passed through graded alcohols, and antigen retrieved with 10 mM citric sodium (pH 6.0) in a steam pressure cooker for 20 min. The slides were then treated with 3% hydrogen peroxide solution in methanol for 10 min to quench endogenous peroxidase activity, blocked with goat serum, and incubated with primary antibodies against YY1 (ab81552, Abcam), followed by incubation with horseradish peroxidase-conjugated secondary antibody for 1 h at RT. Antigen visualization was performed using 3,3′-diaminobenzidine (DAB) chromogen (Vector Laboratories). Slides were counterstained with hematoxylin, dehydrated, and coverslipped with mounting solution (Invitrogen). Whole slides were scanned with a Leica Aperio CS2 slide scanner system (Leica Biosystems). The staining was assessed independently by two pathologists without knowledge of patient characteristics based on the H-score method. The H-score was calculated by adding the percentage of positive cells multiplied by an ordinal value corresponding to the intensity level (none = 0, weak = 1, moderate = 2, strong = 3). H-scores ranged from 0 to 300, and tissue samples were further defined as negative (0–49), weak (50–99), medium (100–199), or positive (200–300).

### Cell cycle analysis

The cell cycle was measured using iodide (PI) staining and flow cytometry analysis. Briefly, genetically edited PC9 cells were collected and washed once with PBS. The cells were then fixed with cold 70% ethanol for 30 min and resuspended in Propidium Iodide (PI)/RNase Staining Solution (Cell Signaling Technology). FACS AriaII cytometer (BD Biosciences) was used for flow cytometry analysis, and the data were processed with FlowJo software.

### Transmission electron microscopy

For transmission electron microscopy (TEM), PC9 cells were treated as indicated, washed twice with PBS, and fixed for 6 h at RT in 2.5% ultrapure glutaraldehyde in PBS. Post-fixation was performed with 1% osmium tetroxide for 90 min at 4 °C, washed four times in PBS, and dehydrated with gradient ethanol solutions (Sinopharm Chemical Reagent) from 50% to 100% in a 10% graded series. Infiltration was carried out using ethanol with propylene oxide (1:1 ratio) for 30 min. The specimens were embedded in Epon 812 resins (Ted Pella), followed by polymerization at 60 °C for 48 h. Ultrathin sections were cut to a thickness of 70 nm by Leica EM UC7 ultramicrotome and stained with saturated uranyl acetate in 50% ethanol, followed by Reynolds lead citrate. Electron microscopy images were obtained using a TEM system (FEI Tecnai G2 Spirit BioTwin).

### RNA extraction and quantitative PCR assay

Total RNA from cells was extracted with RNA Isolation Kit (Vazyme Biotech) and converted to cDNA using the High-Capacity cDNA Reverse Transcription Kit (Invitrogen). Quantitative real-time PCR was performed on a ViiA™ 7 Real-Time PCR System (Applied Biosystems) using ChamQ Universal SYBR qPCR Master Mix (Vazyme Biotech). All samples were normalized to β-actin expression as the endogenous control. At least three biological replicates were included for each condition. The primer sequences used for PCR are listed in Supplementary Table 4.

### RNA-sequencing and analysis

PC9 cells were genetically edited or treated as indicated. Total RNA was purified using the RNeasy Plus kit (Qiagen) following the manufacturer’s instructions. RNA quality was assessed by NanoDrop 8000 (Thermo Fisher Scientific) and agarose gel electrophoresis. Sequencing libraries were prepared using the NEBNext Ultra RNA Library Prep Kit for Illumina (NEB). Clustering of the index-coded libraries on a cBot Cluster Generation System using TruSeq PE Cluster Kit v3-cBot-HS (Illumina), and the library preparations were sequenced on an Illumina Novaseq platform to generate 30 million 150 bp paired-end reads (Novogene). All downstream analyses were based on clean data obtained by removing low-quality reads and reads that contained adapters or ploy-N sequences. The index of the reference genome was built using Bowtie (v2.2.3) [47], and clean reads were aligned to the reference genome using HISAT2 (v2.0.5) [48]. FeatureCounts (v1.5.0-p3) was used to count the reads numbers mapped to each gene [49]. Differential expression analysis was performed using DESeq2 (v1.20.0) R package [50]. Transcripts with adjusted *P*-values of <0.05 were assigned as differentially expressed genes.

### ATAC sequencing

The assay for transposase-accessible chromatin using sequencing (ATAC-seq) was performed as previously described [51]. First, a total of 5 × 10^4^ cells were harvested in ice-cold washing buffer (10 mM Tris-HCl pH 7.4, 10 mM NaCl, 3 mM MgCl_2_, 0.1% IGEPAL CA-630). Cell pellets were resuspended in Tn5 transposition reaction mix containing 25 µL 2× TD Buffer, 2.5 µL Tn5 Transposes (Illumina), and 22.5 µL nuclease-free water, and incubated at 37 °C for 30 min. DNA was purified with MinElute PCR Purification Kit (Qiagen). Eluted DNA was barcoded and amplified using NEBNext Q5 Hot Start HiFi PCR Master Mix (#M0543L, New England Labs) in a total volume of 50 µL with the following PCR program: 72 °C for 3 min; 98 °C for 30 s; 12 cycles of 98 °C for 30 s, 63 °C for 30 s and 72 °C for 3 min; 72 °C for 5 min. The libraries were purified with QIAquick PCR Purification Kit (Qiagen) and sequenced on the Illumina HiSeq with 50-bp paired-ends. Reads were aligned to the human reference genome (GRCh37/hg19) using BWA with standard parameters [52], and filtered for high-quality (MAPQ ≥ 13), non-mitochondrial, and properly paired reads (longer than 18 nt). MACS2 (v 2.2.6) was used to call peaks using the parameter “-q 0.05 -g hs --nomodel --shift -75 --extsize 150” [53]. Peaks with an FDR of lower than 5% were saved to detect chromosomal regions for further analysis. Bigwig files were generated from the filtered BAM files using deepTools2 bamCoverage with the option “--normalizeUsing RPKM” [54]. Peak annotation was performed with HOMER annotatePeaks.pl [55]. Heatmaps and average profile plots were generated using deepTools plotHeatmap. Coverage tracks were visualized using the trackViewer R package [56].

### CUT&Tag analysis

Cleavage under targets & tagmentation (CUT&Tag) was performed using the Hyperactive In-Situ ChIP Library Prep Kit for Illumina (TD901, Vazyme Biotech) following the manufacturer’s instructions. Briefly, cells were collected into a low absorbing tube, washed twice in 500 μL Wash Buffer, and incubated with concanavalin A-coated magnetic beads for 10 min at RT. The bead-bound cells were resuspended in 50 μL Antibody Buffer, followed by incubation with 1 μg of primary antibody at 4 °C overnight with slow rotation. The supernatant was removed, and cells were incubated with a secondary antibody in a Dig-wash Buffer for 1 h at room temperature. Cells were washed three times with Dig-wash Buffer and incubated with 0.04 µM Hyperactive pG-Tn5 Transposase adapter mix prepared in Dig-300 buffer. Cells were washed three times with Dig-300 buffer to remove unbound pG-Tn5 and resuspended in 300 µL Tagmentation Buffer to incubate for 1 h at 37 °C. The reaction was terminated, and DNA was extracted using the phenol-chloroform-isoamyl alcohol method. PCR was performed to amplify the libraries under the following cycling conditions: 72 °C for 5 min, 98 °C for 30 s, 20 cycles of 98 °C for 10 s, and 60 °C for 30 s, followed by a final extension at 72 °C for 1 min and holding at 4 °C. PCR products were then purified with DNA Clean Beads. After quantification and quality control, DNA libraries were sequenced on an Illumina NovaSeq platform. The following antibodies were used: anti-rabbit IgG (#2729, Cell Signaling Technology), YY1 (ab109237, Abcam). Peaks were called using SEACR (v1.1) and MACS2 (v2.2.6) [53, 57]. Subsequent analyses were the same as those for the ATAC-seq pipeline.

### In vivo studies

To evaluate the function of YY1 in vivo, 1 × 10^6^ PC9 cells with YY1 genetic editing were mixed with Matrigel (BD Biosciences) and subcutaneously implanted in the dorsal flank of BALB/c nude mice (5 weeks of age). When tumor sizes reached approximately 200–300 mm^3^, animals were randomized into vehicle and treatment groups (8 mice each). For the treatment of tumor-bearing mice, erlotinib was administered at a dose of 5 mg/kg/day via gavage. Tumor volumes were recorded by blind measurements with a caliper and calculated as length × width^2^ × 0.52. The Institutional Animal Care and Use Committee (IACUC) of Ren Ji Hospital approved all animal protocols.

### Statistical analysis

The sequencing data were deposited in the NCBI BioProject database under the accession number PRJNA973187. TCGA data were downloaded from the UCSC Xena Explorer and processed as previously described [58]. The Kaplan–Meier method with log-rank test was used for survival analysis. Pathway enrichment analysis of differentially expressed genes was performed using the clusterProfiler R package (v3.18.0) and Metascape (http://metascape.org) [59]. Gene set enrichment analysis was performed using the GSEA software (v4.1.0). In all experiments, comparisons between two groups were based on two-sided Student’s *t*-tests. Pearson’s correlation coefficient was used to measure the linear correlation between two variables. Graphics and statistics were generated using GraphPad Prism (v8.0) or R (v4.1.0). *P*-values of <0.05 were considered statistically significant.

## Supplementary information

supplementary Figure

Supplementary Tables

original data

## Data Availability

Original images of immunoblotting are provided in the Supplementary information. All data and materials generated in this study are available upon request from the corresponding authors.
