# Supplementary material for: YY1 downregulation underlies therapeutic response to molecular targeted agents
Source: Cell Death Dis. 2024 Nov 27;15(11):862. doi: 10.1038/s41419-024-07239-8 (PMC11603335; doi:10.1038/s41419-024-07239-8)
Supplement: Supplementary file 1 — supplementary Figure [file 41419_2024_7239_MOESM1_ESM.pdf]

Supplementary Figure 1.

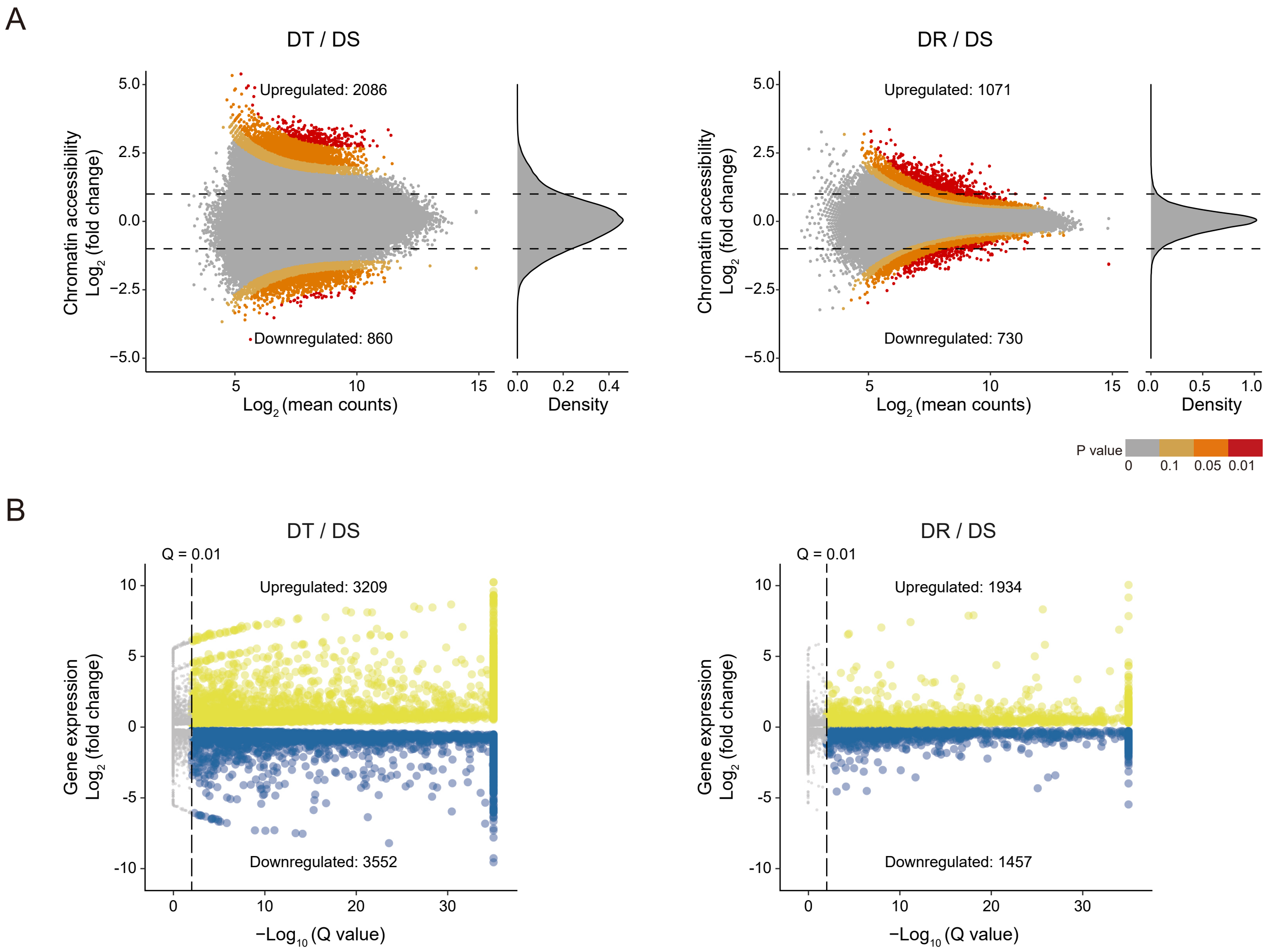

Supplementary Figure 1.
A. Volcano plot showing differential chromatin accessibility at drug-tolerant persister state (DT) or acquired drug-resistant state (DR) compared to the initial drug-sensitive state (DS).
B. Volcano plot showing differentially expressed genes at drug-tolerant persister state (DT) or acquired drug-resistant state (DR) compared to the initial drug-sensitive state (DS). Q value represents adjusted *P* value.

Supplementary Figure 2.

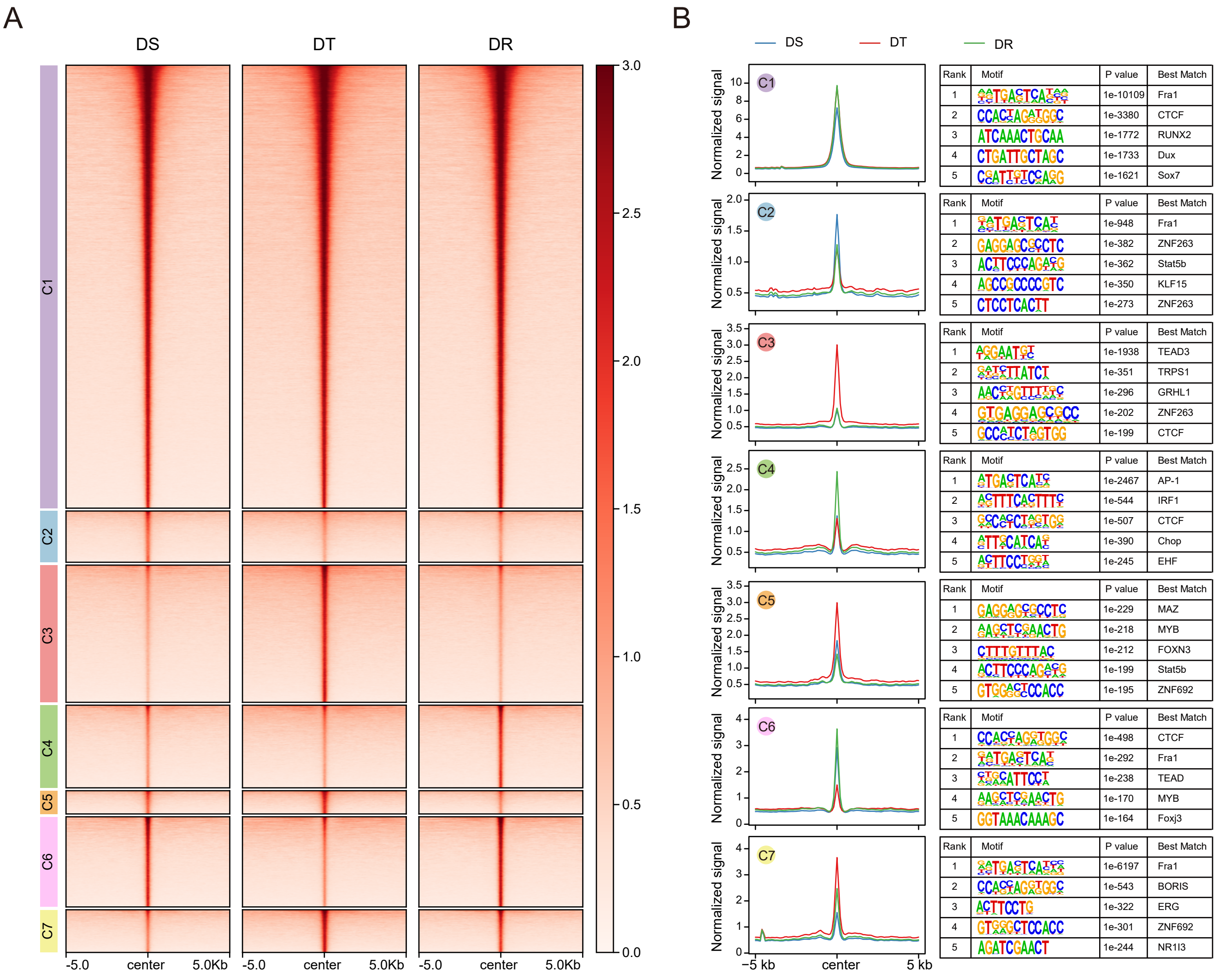

Supplementary Figure 2.

A. Heatmaps depicting ATAC-seq peak signal intensities at the initial drug-sensitive state (DS), drug-tolerant persister state (DT), and acquired drug-resistant state (DR). Seven clusters (C1-C7) of chromatin accessible loci were identified.

B. Transcription factor motif analysis of seven ATAC-seq peak clusters.

Supplementary Figure 3.

A

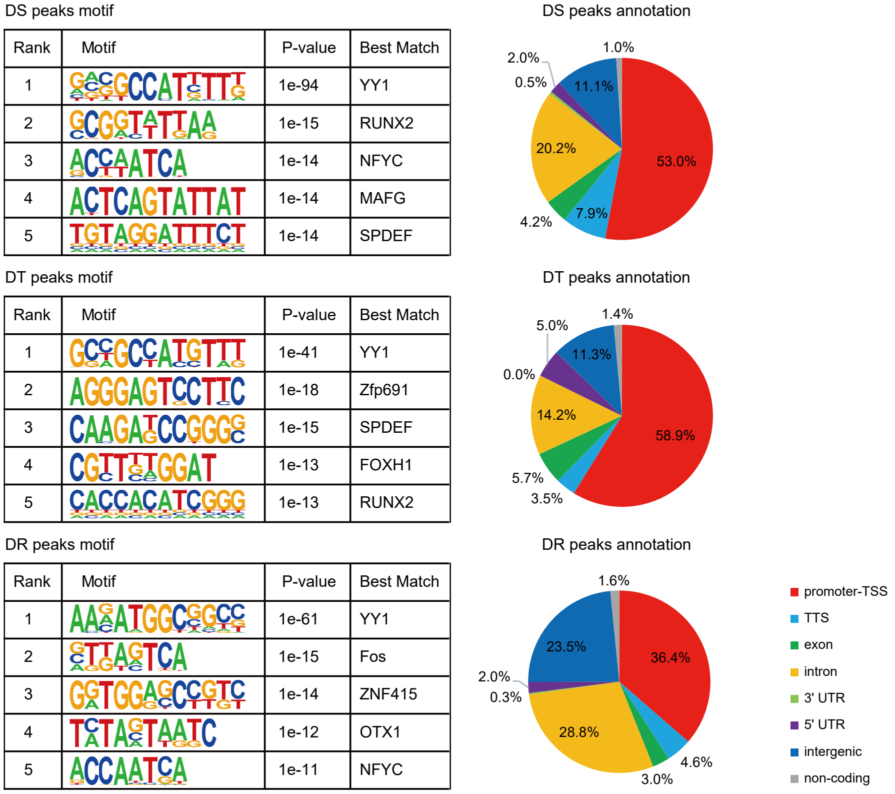

B

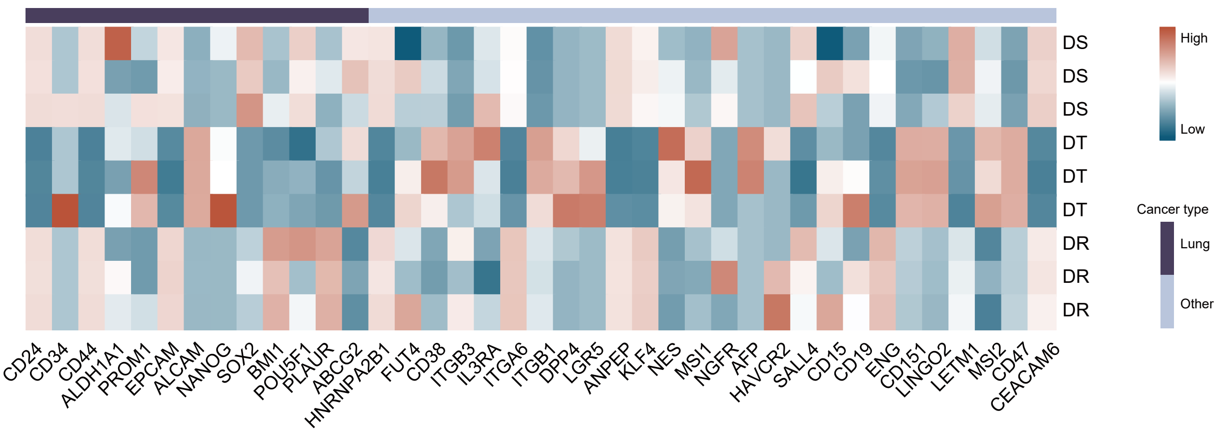

Supplementary Figure 3.

A. Motif analysis of YY1 CUT&Tag peaks at the initial drug-sensitive state (DS), drug-tolerant persister state (DT), and acquired drug-resistant state (DR). Pie graph displaying the distribution of YY1 CUT&Tag peaks annotation at the initial drug-sensitive state (DS), drug-tolerant persisterstate (DT), and acquired drug-resistant state (DR). TSS: transcriptional start site. TTS: transcription termination site. UTR: untranslated region.

B. Heatmaps depicting gene expression of putative cancer stem cell markers at the initial drug-sensitive state (DS), drug-tolerant persister state (DT), and acquired drug-resistant state (DR).

Supplementary Figure 4.

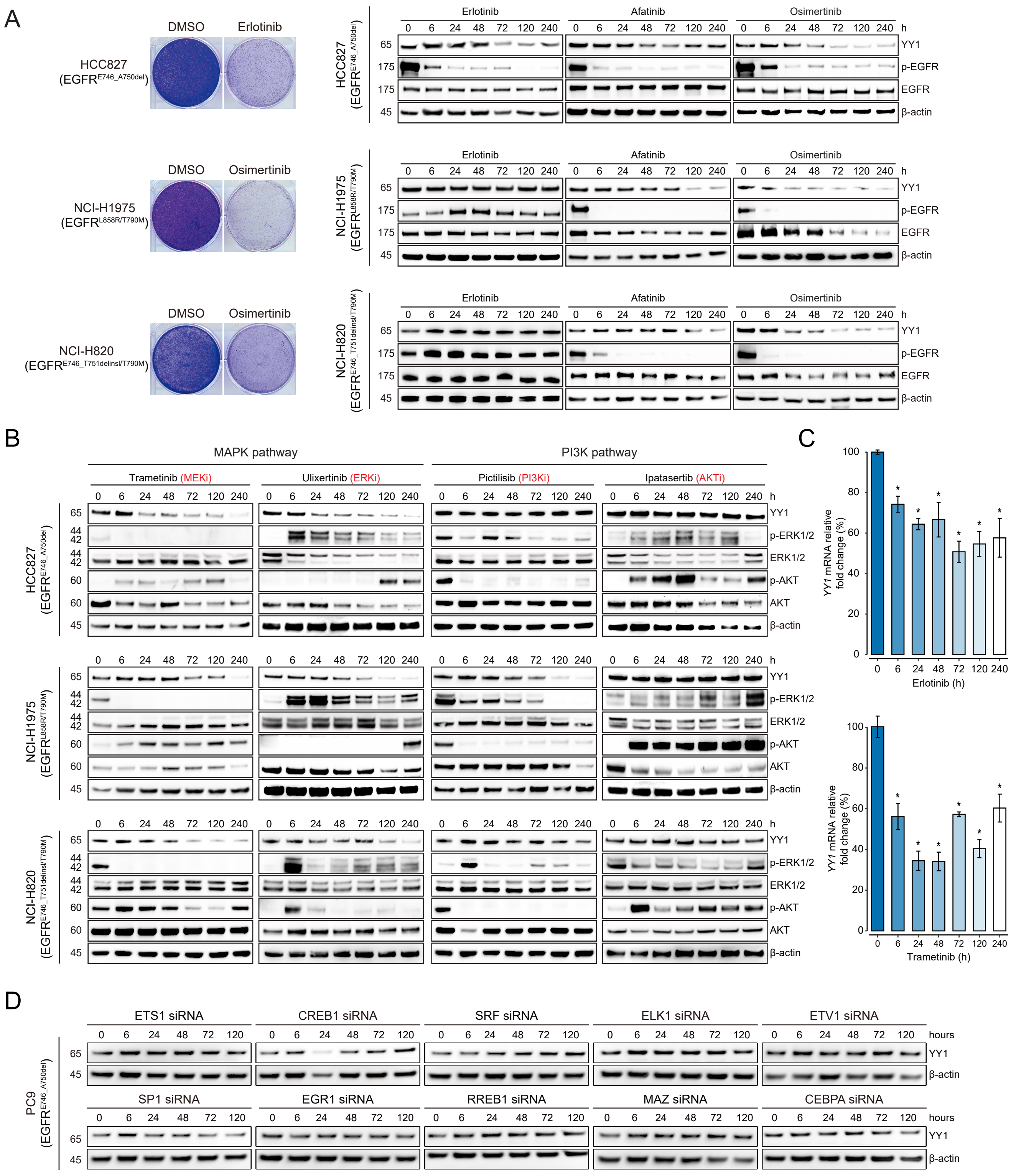

Supplementary Figure 4.

A. HCC827, NCI-H1975 and NCI-H820 cells were treated with erlotinib, afatinib, or osimertinib in a time course manner, and the indicated proteins were analyzed by immunoblotting. Several EGFR inhibitors response was assessed by crystal violet staining.

B. HCC827, NCI-H1975 or NCI-H820 cells were treated with MAPK pathway inhibitors (trametinib and ulixertinib) or PI3K pathway inhibitors (pictilisib and ipatasertib) in a time course manner, and the indicated proteins were analyzed by immunoblotting.

C. PC9 cells were treated with erlotinib or trametinib in a time course manner, and the relative expression of YY1 mRNA was measured by quantitative PCR analysis.

D. Transcription factors related to the MAPK pathway were knocked down in PC9 cells with siRNAs, and YY1 protein was analyzed by immunoblotting.

Supplementary Figure 5.

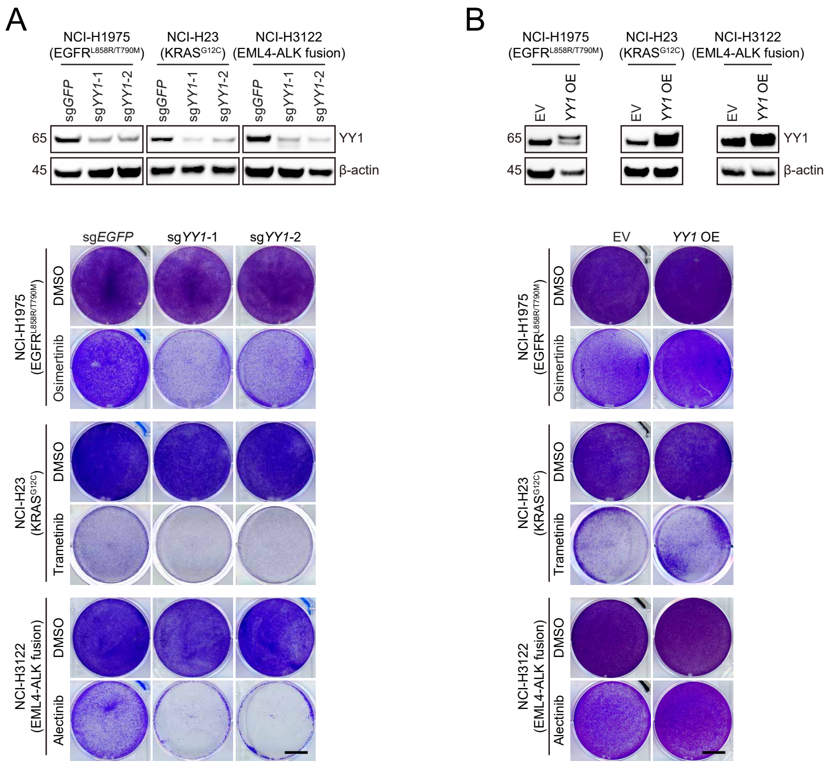

Supplementary Figure 5.

A. *YY1* was knocked out in NCI-H1975, NCI-H23 or NCI-H3122 cells with two independent sgRNAs using the CRISPR-Cas9 system, and YY1 protein was analyzed by immunoblotting. EGFR inhibitor response upon *YY1* depletion was assessed by crystal violet staining. Scale bar represents 10 mm.

B. *YY1* was overexpressed in NCI-H1975, NCI-H23 or NCI-H3122 cells, and YY1 protein was analyzed by immunoblotting. EGFR inhibitor response upon *YY1* overexpression was assessed by crystal violet staining. Scale bar represents 10 mm.

Supplementary Figure 6.

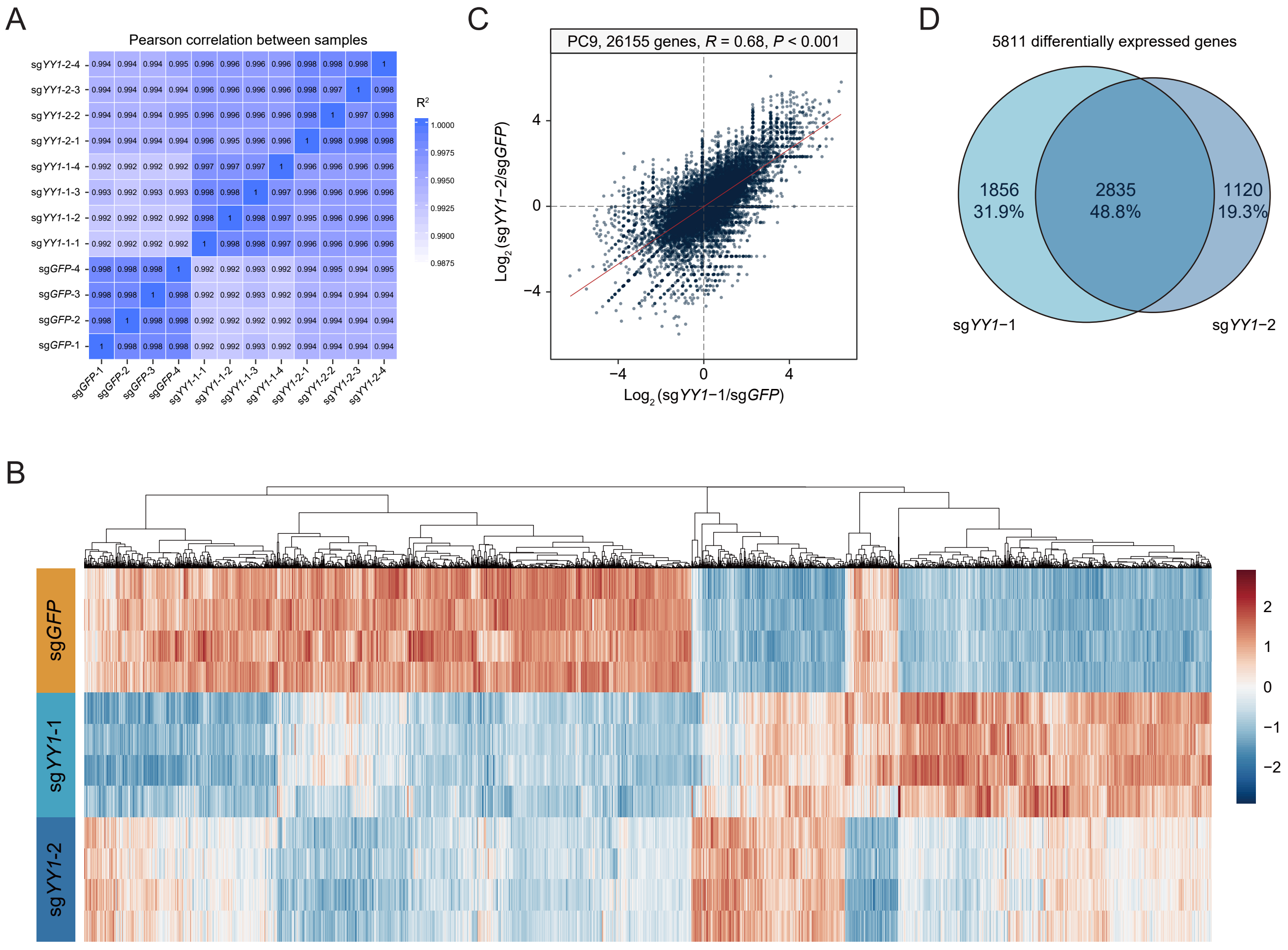

Supplementary Figure 6.

A. Heatmap illustrating pairwise correlation of RNA-seq data upon *YY1* depletion using two independent sgRNAs in PC9 cells.

B. Heatmap depicting differentially expressed genes upon *YY1* depletion using two independent sgRNAs in PC9 cells.

C. Correlation estimation of fold changes induced by *YY1* depletion using two independent sgRNAs in PC9 cells at the transcriptome-wide scale. Pearson correlation coefficient (r) and *P* value are shown.

D. Venn diagram illustrating the proportion of overlapping differentially expressed genes upon *YY1* depletion using two independent sgRNAs in PC9 cells.

Supplementary Figure 7.

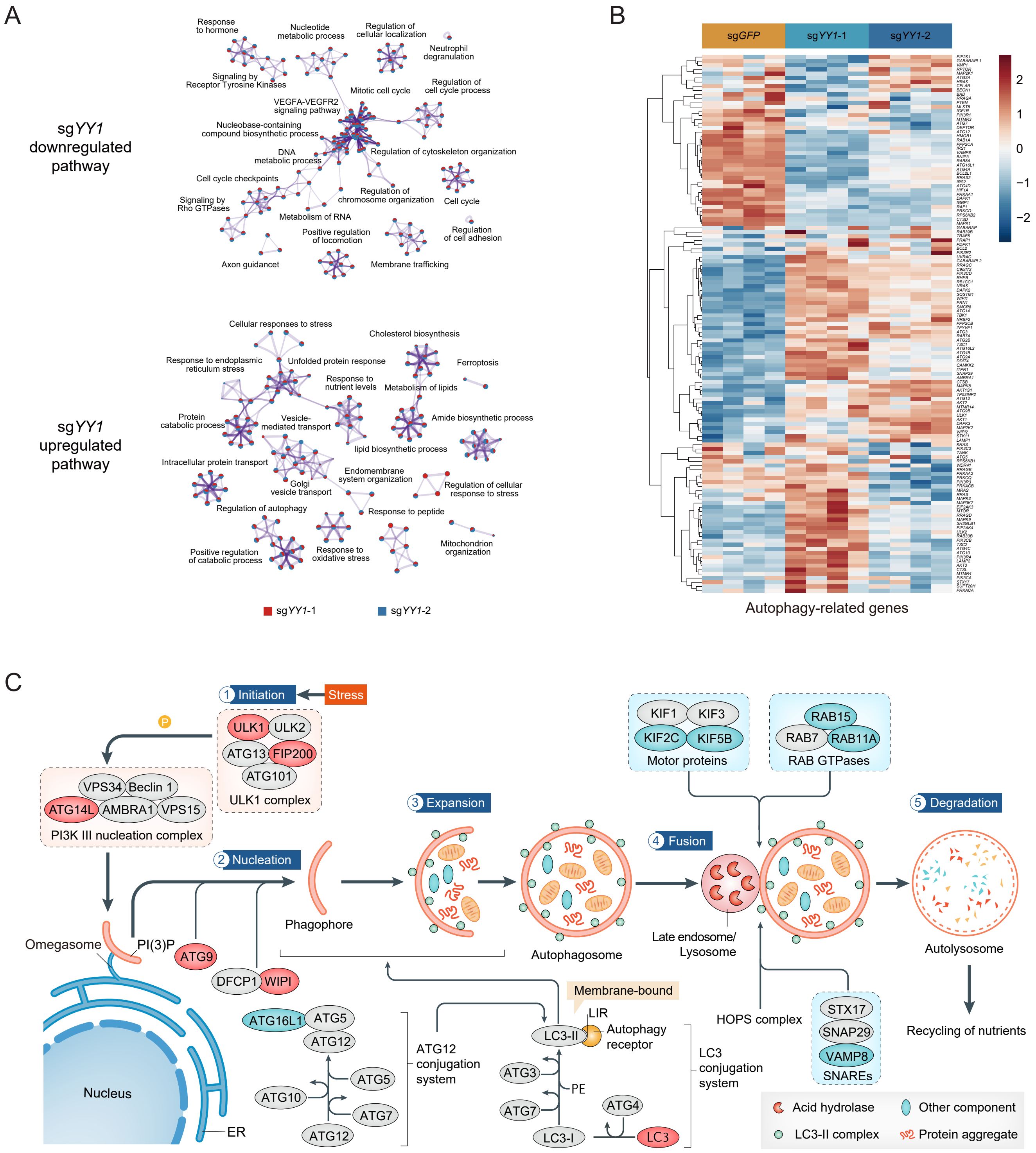

Supplementary Figure 7.

A. Cytoscape network visualization on Metascape enriched terms of differentially expressed genes upon *YY1* depletion using two independent sgRNAs in PC9 cells. Each node, colored based on the sgRNAs, represents an enriched term and is connected by edges with similar terms.

B. Heatmap depicting differentially expressed autophagy-related genes upon *YY1* depletion using two independent sgRNAs in PC9 cells.

C. A schematic diagram illustrating the autophagic cascade and the dispersion of differentially expressed autophagy-related genes upon *YY1* depletion in PC9 cells.
