# Supplementary material for: YY1 downregulation underlies therapeutic response to molecular targeted agents
Source: Cell Death Dis. 2024 Nov 27;15(11):862. doi: 10.1038/s41419-024-07239-8 (PMC11603335; doi:10.1038/s41419-024-07239-8)

Fig 2.A

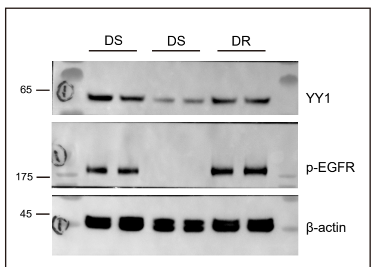

Fig 2.B

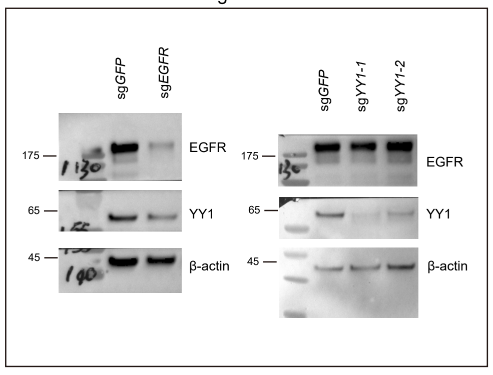

Fig 2.F

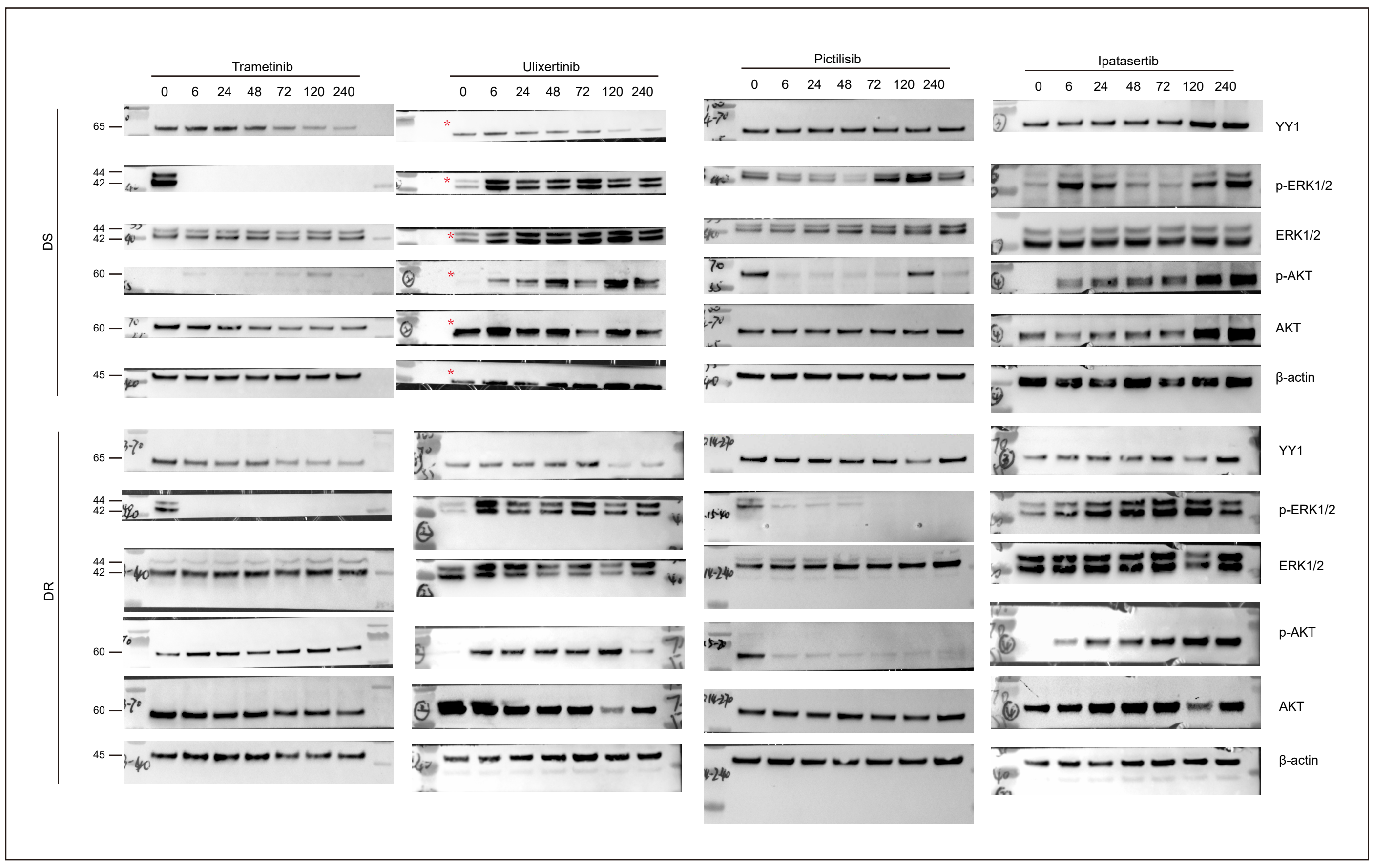

Fig 2.C

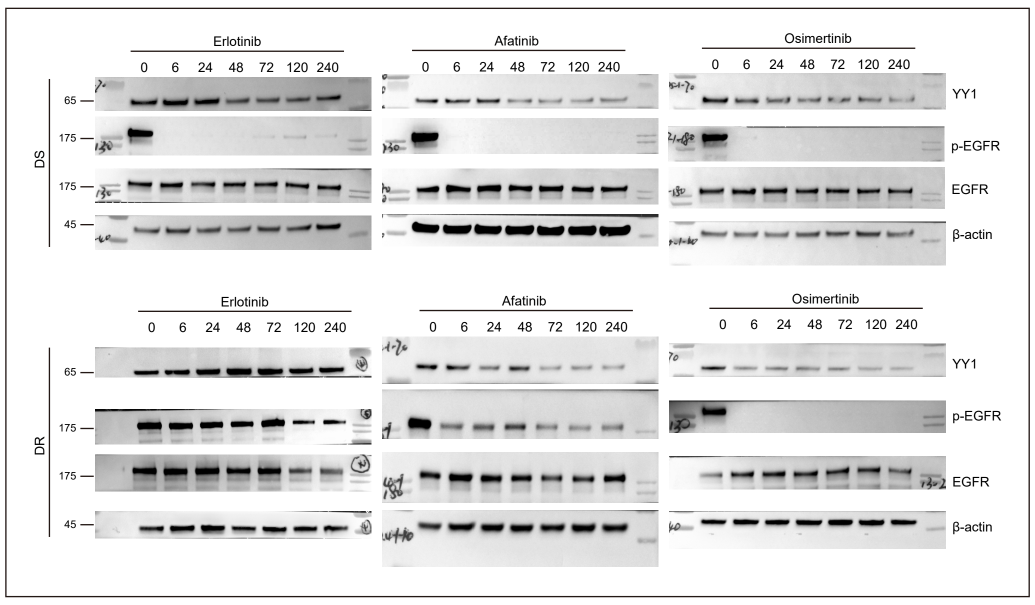

Fig 3.D

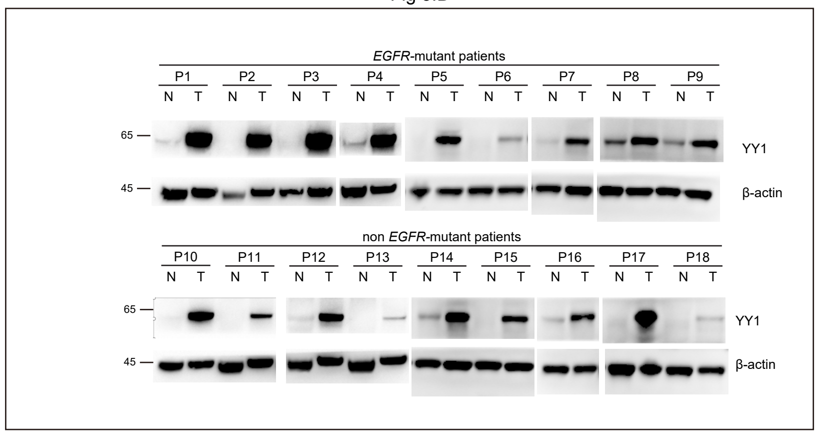

Fig 3.C

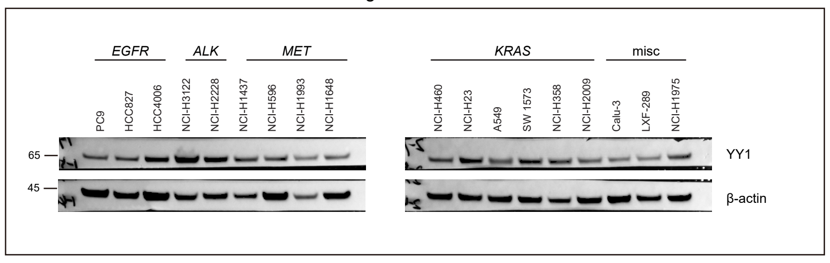

Fig 3.E

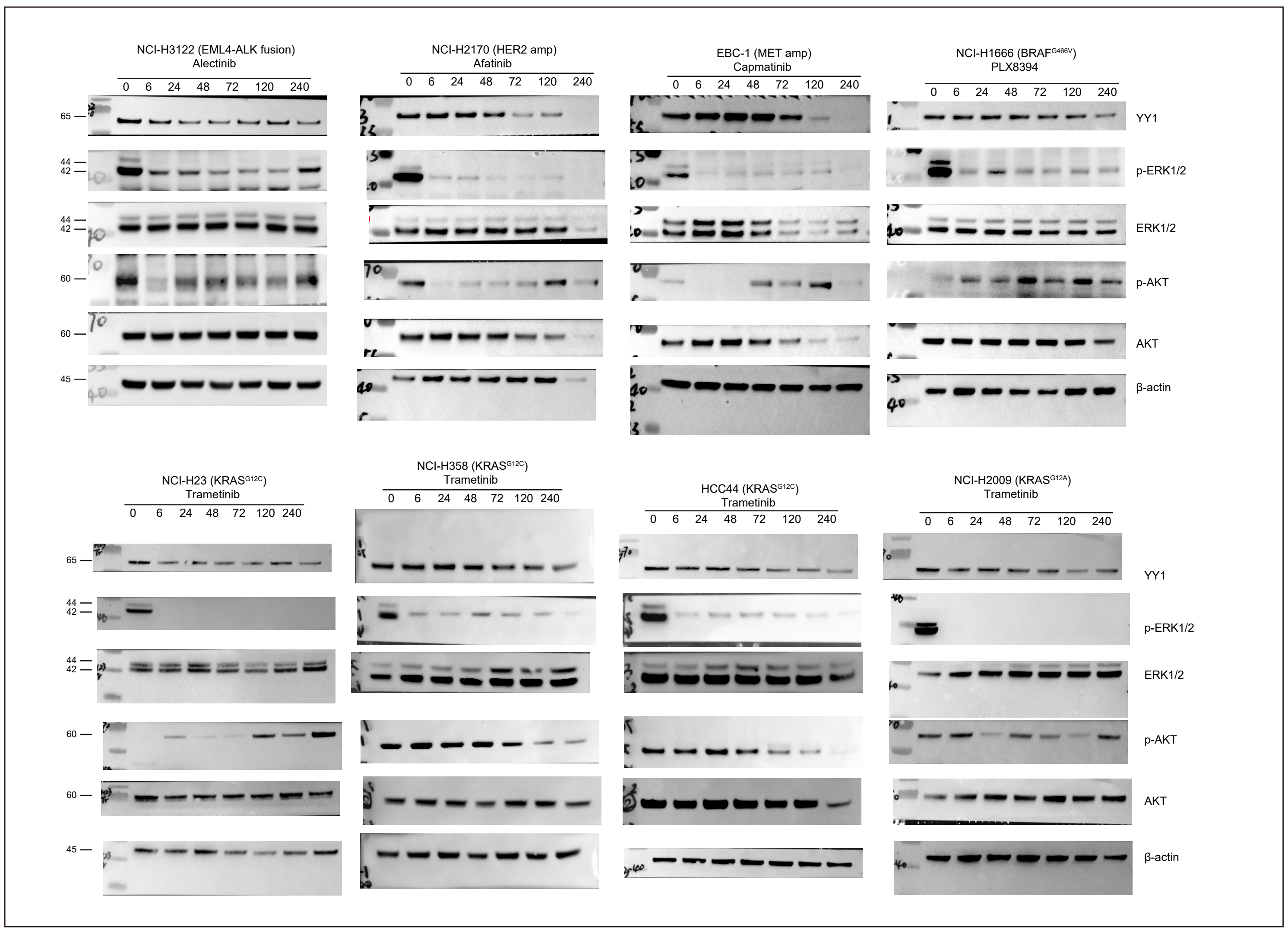

Fig 3.F

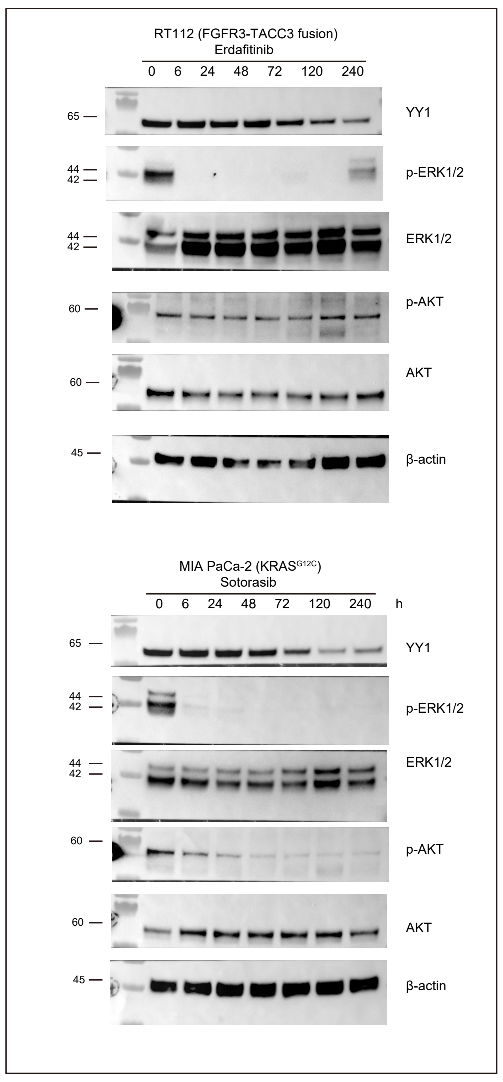

Fig 4.A

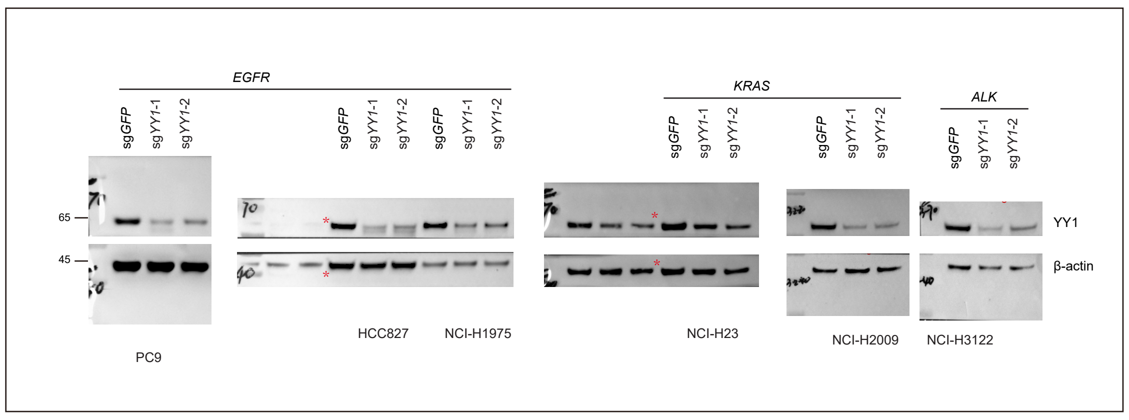

Fig 4.D

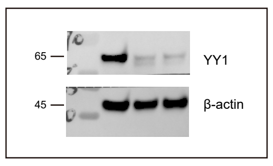

Fig 4.E

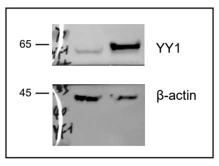

Fig 5.E

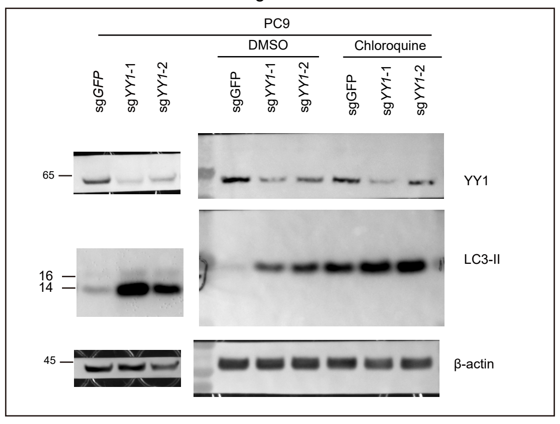

Fig 5.F

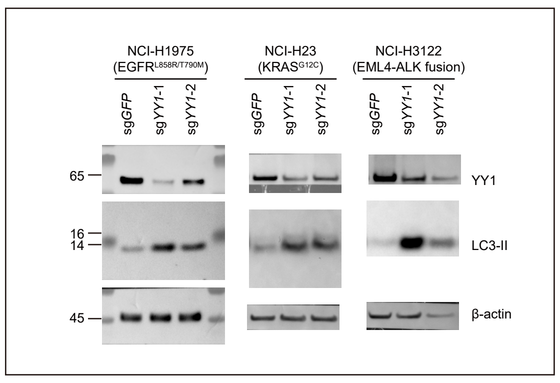

Supplementary Fig 4.A

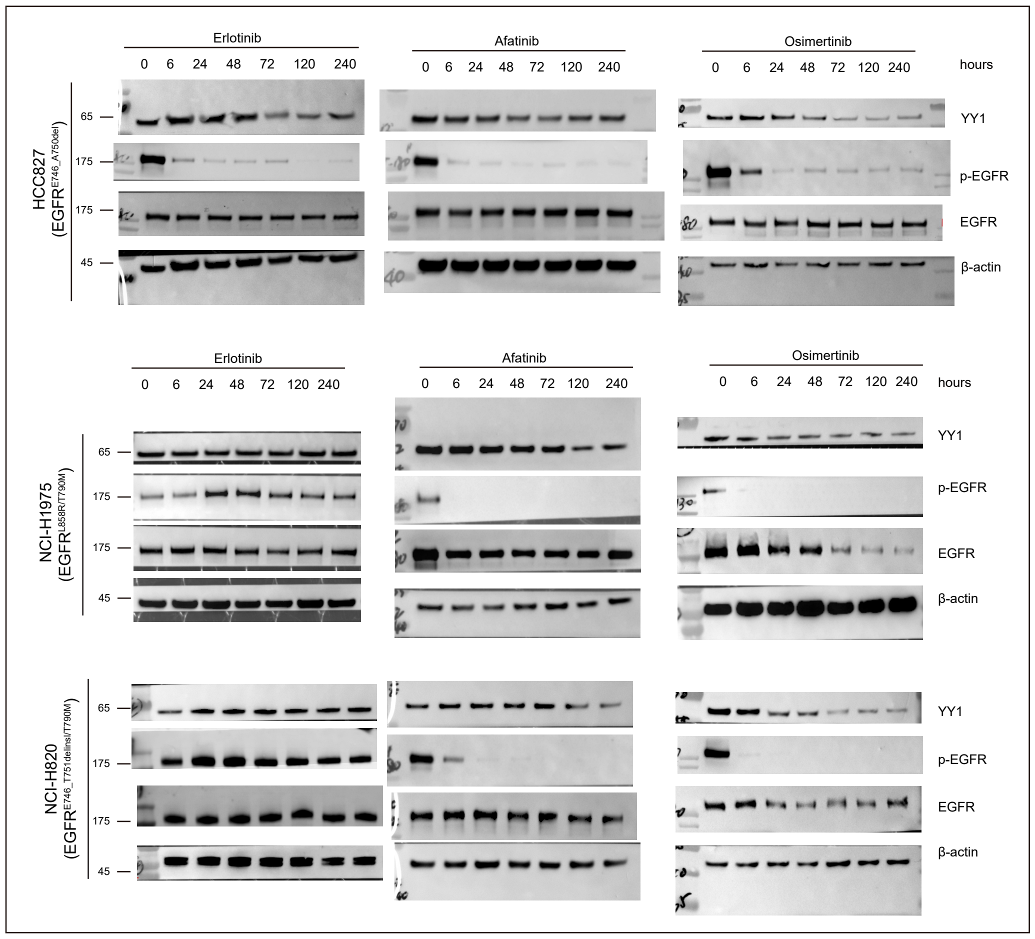

Supplementary Fig 4.B

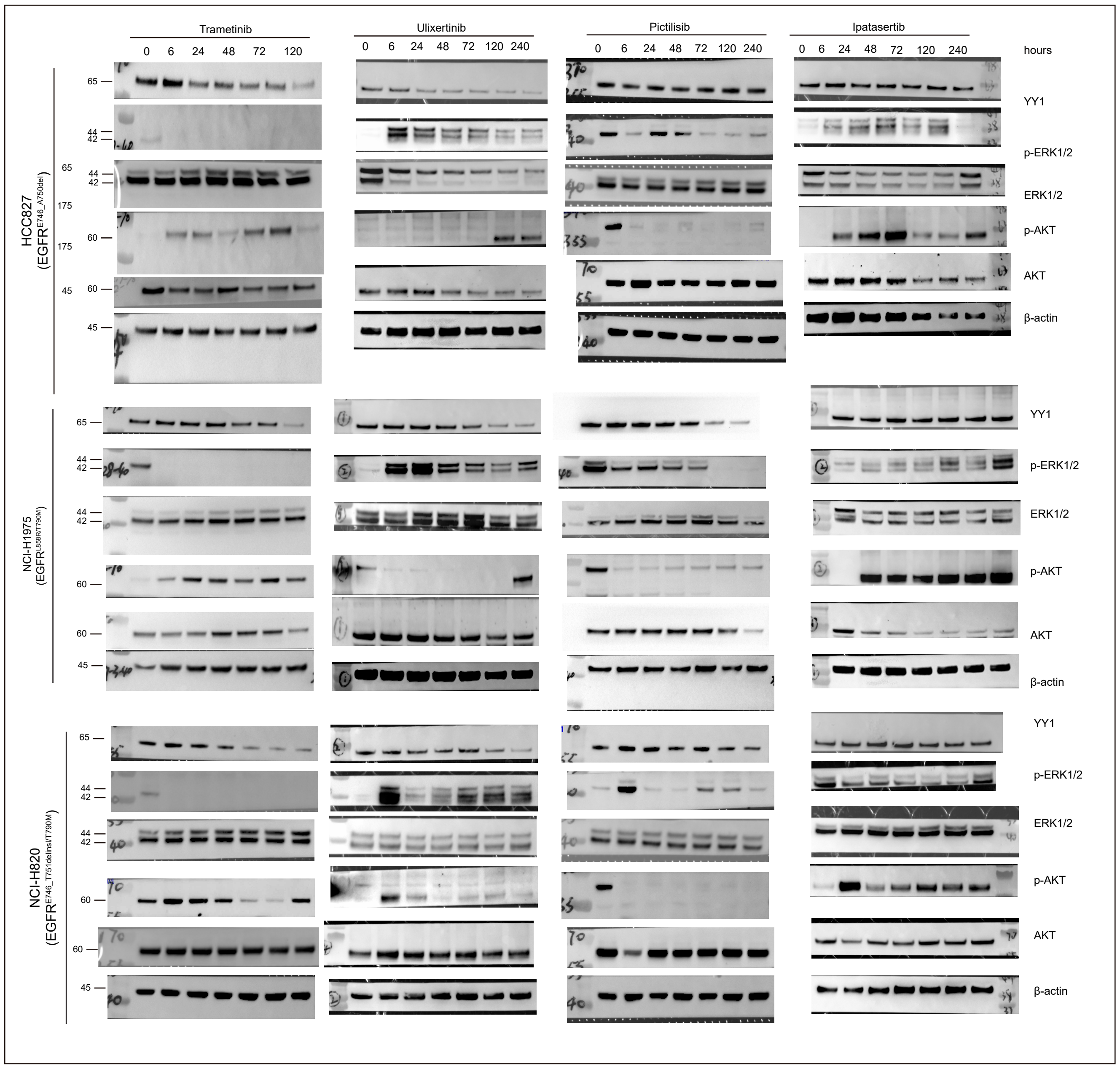

Supplementary Fig 5.A

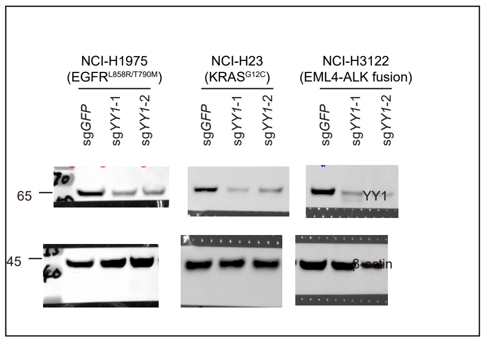

Supplementary Fig 5.B

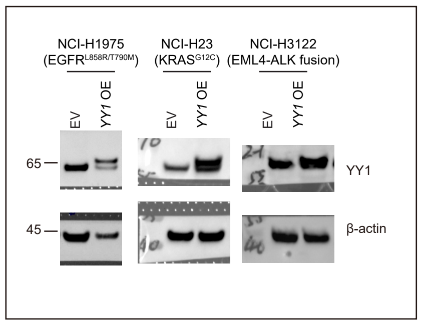

Supplementary Fig 4.D

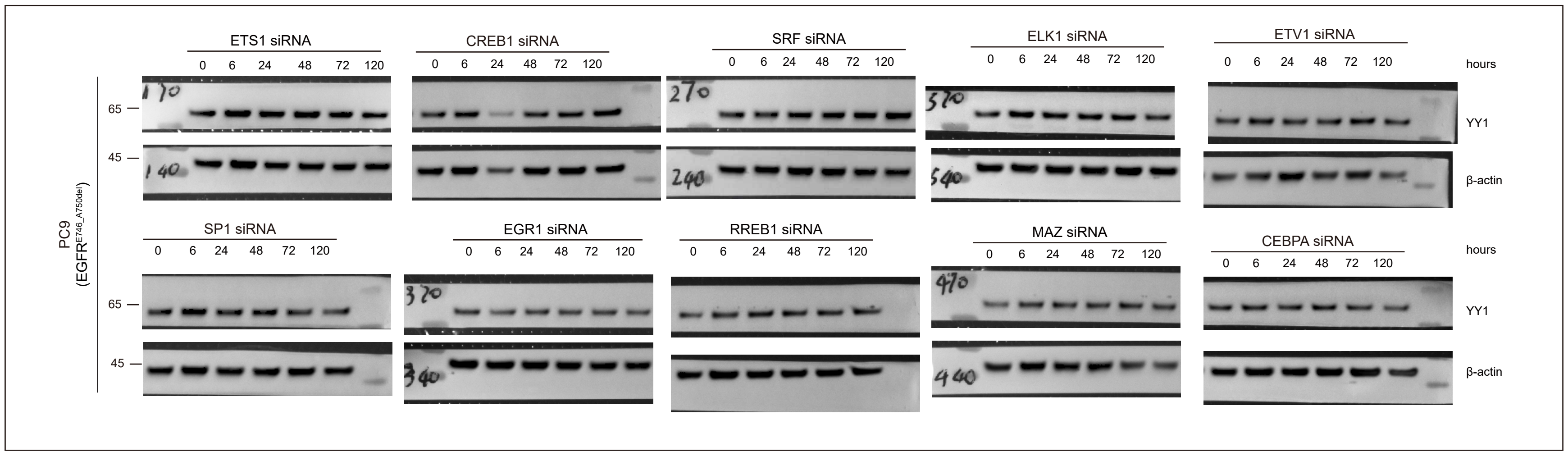

Supplement: Supplementary file 3 — original data [file 41419_2024_7239_MOESM3_ESM.pdf]
